# A scoping review exploring women’s experiences of cardiometabolic pregnancy complications and future cardiovascular health implications

**DOI:** 10.1038/s44325-026-00107-8

**Published:** 2026-03-23

**Authors:** Wentong Xu, Monique Wisnewski, Caroline Kindsvater, Helena Teede, Lisa J. Moran, Sarah Lang

**Affiliations:** 1 Monash Centre for Health Research and Implementation, School of Clinical Sciences, Monash University, Clayton, VIC Australia; 2 Monash Endocrine and Diabetes Units, Monash Health, Clayton, VIC Australia; 3 Monash Victorian Heart Institute, Monash University, Clayton, VIC Australia

**Keywords:** Diseases, Endocrinology, Health care, Medical research, Risk factors

## Abstract

Women who develop cardiometabolic pregnancy complications including gestational diabetes mellitus (GDM), hypertensive disorders of pregnancy (HDP), fetal growth restriction (FGR) and preterm birth (PTB) are at increased risk of type 2 diabetes and cardiovascular disease. This scoping review explores the extent, range and nature of qualitative research investigating experiences of women at-risk of or diagnosed with cardiometabolic pregnancy complications. Original research (*n* = 623) and review (*n* = 66) articles with qualitative data from women, partners, healthcare professionals and community and professional stakeholders were included. Studies involving women with GDM (*n* = 342, 49.6%), HDP (*n* = 163, 23.7%) and FGR (*n* = 17, 2.5%) primarily explored lifestyle, medical or pharmacotherapy interventions during pregnancy, whereas studies regarding PTB (*n* = 198, 28.7%) primarily explored psychosocial health postpartum. Risk of future diabetes or cardiovascular disease were prevalent concepts in GDM and HDP literature. Extensive qualitative research exists relating to maternal cardiometabolic health. Understanding women’s experiences may guide cardiovascular disease prevention and screening initiatives.

## Introduction

Women who experience cardiometabolic pregnancy complications, including gestational diabetes mellitus (GDM), hypertensive disorders of pregnancy (HDP), fetal growth restriction (FGR) and spontaneous preterm birth (PTB), are at increased risk of future type 2 diabetes (T2DM) and cardiovascular disease (CVD)^1–4^. GDM is impaired glucose metabolism beginning in pregnancy^5^. HDP includes gestational hypertension (systolic blood pressure >140 mmHg or diastolic blood pressure >90 mmHg occurring after 20 weeks’ gestation) and pre-eclampsia (gestational hypertension with end-organ dysfunction) with its various complications^6^. FGR is when a fetus does not reach its genetically predetermined potential growth^7^, distinct from constitutionally small for gestational age (SGA) fetuses. FGR is difficult to detect antenatally with SGA often used as a research proxy^8,9^. Spontaneous PTB is birth before 37 weeks’ gestation that was not medically induced, i.e. following spontaneous preterm labour or preterm premature rupture of membranes (PPROM)^10^.

While GDM, HDP, FGR and PTB have distinct pathophysiological processes, the metabolic stress and vascular changes of pregnancy that predispose patients to these conditions underpin a shared risk of future cardiovascular complications^11^. These conditions can be considered sex-specific risk factors for the development of diabetes and hypertension, compounded by the increased incidence of traditional cardiovascular risk factors postpartum^2,12,13^. While recommendations for postpartum screening and lifestyle changes to optimize cardiometabolic health exist for GDM^14,15^ and HDP^16^, these are often poorly implemented^17–19^. Additionally, while PTB and FGR describe fetal outcomes, they are increasingly recognized as risk factors for T2DM and CVD, acknowledging their complex multi-causal aetiologies, including as sequelae of maternal pathophysiological processes such as GDM and HDP^20^. Together these cardiometabolic complications have implications on women’s long-term cardiovascular health, noting that poor antenatal control increases the risk of adverse outcomes^21,22^. The American Heart Association (AHA) identifies these pregnancy complications as opportunities for CVD prevention and risk stratification^11^.

There is a drive to increase research and develop strategies to optimize women’s pregnancy care and support T2DM and CVD primary prevention^11^. Qualitative research provides insights into women’s experiences, with the potential to tailor care and develop disease prevention efforts that reflect the complexity of women’s healthcare around pregnancy. Synthesizing qualitative research will capture global perspectives of women’s lived experiences as well as perspectives of healthcare providers and other stakeholders, allowing researchers to leverage knowledge and identify opportunities for future research. This scoping review aims to explore the extent, range and nature of qualitative research studies investigating women at-risk of or diagnosed with GDM, HDP, FGR and/or spontaneous PTB during pregnancy and postpartum.

## Results

### General characteristics of the studies

**Fig. 1:**
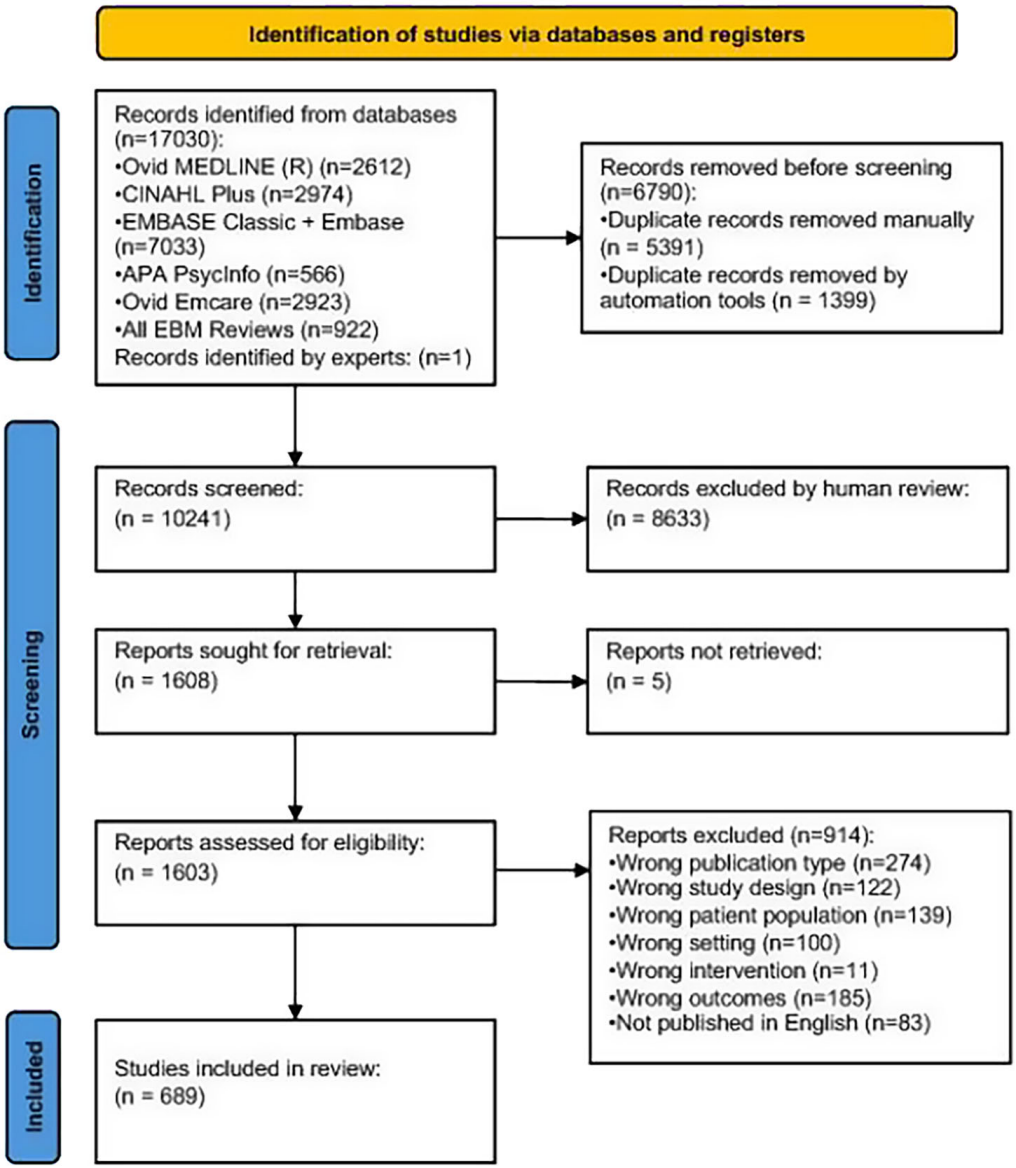
PRISMA diagram. This diagram shows the systematic process we followed to identify eligible papers from our search.

The original search from inception to 2022 returned 11557 studies, while the update from 2022 to February 2025 returned an additional 5473 studies. 5391 duplicate studies were removed manually, and 1399 duplicate studies were removed by automation tools. One additional expert identified study was included. In total, 10,241 articles were screened by title and abstract and 1608 studies were screened by full text (Fig. 1). Reasons for exclusion were wrong publication type (*n* = 274), wrong study design (*n* = 122), wrong patient population (*n* = 139), wrong setting (*n* = 100), wrong intervention (*n* = 11), wrong outcomes (*n* = 185), not published in English (*n* = 83) and no full text available (*n* = 5) (Fig. 1). Overall, 689 articles met the inclusion criteria, including original research studies involving women with one cardiometabolic condition (*n* = 600), original research studies involving mixed sample populations (*n* = 23) and reviews (*n* = 66), including one review on multiple cardiometabolic complications. The number of included studies and their key characteristics are outlined in Table 1.

**Table 1:** Number of included studies and characteristics

Condition of interest - literature type	Year of publication	Region	Data collection method	Timing of assessment	Total participants^f^
GDM - Original Research (*n* = 299^a^)	<2000 (*n* = 1) 2000–2009 (*n* = 10) 2010–2019 (*n* = 120) >/=2020 (*n* = 168)	Europe (*n* = 97) North America (*n* = 76) South America (*n* = 5) Oceania (*n* = 54) Asia (*n* = 51) Africa (*n* = 16)	Interviews (*n* = 187) Focus groups (*n* = 28) Surveys (*n* = 9) Mixed methods (*n* = 75)	Pregnancy (*n* = 143) Postpartum (*n* = 124) Pregnancy & Postpartum (*n* = 32)	Women (*n* = 7794) Healthcare professionals (*n* = 2209) Partners (*n* = 54) Community stakeholders (*n* = 48) Professional stakeholders (*n* = 150)
GDM - Review Articles (*n* = 42^b^)	2010–2019 (*n* = 11) >/=2020 (*n* = 31)	Europe (*n* = 15) North America (*n* = 4) Oceania (*n* = 13) Asia (*n* = 7) Africa (*n* = 3)	Qualitative systematic review (*n* = 18) Systematic review (*n* = 17) Scoping review (*n* = 4) Integrative review (*n* = 1) Interpretive review (*n* = 1) Umbrella review (*n* = 1)	N/A	Total number of qualitative/mixed methods articles synthesized (*n* = 787)
HDP – Original Research (*n* = 150^c^)	<2000 (*n* = 3) 2000–2009 (*n* = 7) 2010–2019 (*n* = 35) >/=2020 (*n* = 105)	Europe (*n* = 52) North America (*n* = 34) South America (*n* = 8) Oceania (*n* = 13) Asia (*n* = 15) Africa (*n* = 26) Multiregional (*n* = 2)	Interviews (*n* = 98) Focus groups (*n* = 8) Surveys (*n* = 5) Social media content analysis (*n* = 2) Mixed methods (*n* = 37)	Pregnancy (*n* = 72) Postpartum (*n* = 67) Pregnancy & Postpartum (*n* = 10) Preconception & Pregnancy (*n* = 1)	Women (*n* = 5751) Healthcare professionals (*n* = 1224) Partners (*n* = 80) Community stakeholders (*n* = 62) Professional stakeholders (*n* = 34)
HDP – Review Articles (*n* = 13^b^)	2010–2019 (*n* = 2) >/=2020 (*n* = 11)	Europe (*n* = 4) North America (*n* = 2) South America (*n* = 2) Oceania (*n* = 2) Asia (*n* = 3)	Qualitative systematic review (*n* = 5) Scoping review (*n* = 5) Systematic review (*n* = 3)	N/A	Total number of qualitative/mixed methods articles synthesized (*n* = 141)
FGR – Original Research (*n* = 17^d^)	<2000 (*n* = 1) 2000–2009 (*n* = 2) 2010–2019 (*n* = 8) >/=2020 (*n* = 6)	Europe (*n* = 8) North America (*n* = 5) South America (*n* = 1) Oceania (*n* = 1) Asia (*n* = 1) Africa (*n* = 1)	Interviews (*n* = 8) Surveys (*n* = 1) Mixed methods (*n* = 8)	Pregnancy (*n* = 6) Postpartum (*n* = 10) Pregnancy & Postpartum (*n* = 1)	Women (*n* = 394) Healthcare professionals (*n* = 29) Partners (*n* = 23) Professional stakeholders (*n* = 34)
PTB – Original Research (*n* = 185^e^)	<2000 (*n* = 11) 2000–2009 (*n* = 25) 2010–2019 (*n* = 65) >/=2020 (*n* = 84)	Europe (*n* = 58) North America (*n* = 67) South America (*n* = 10) Oceania (*n* = 12) Asia (*n* = 17) Africa (*n* = 21)	Interviews (*n* = 129) Focus groups (*n* = 13) Surveys (*n* = 6) Case studies (n = 2) Meetings (*n* = 1) Mixed methods (*n* = 34)	Pregnancy (*n* = 39) Postpartum (*n* = 134) Pregnancy & Postpartum (*n* = 12)	Women (*n* = 6634) Healthcare professionals (*n* = 647) Partners (*n* = 785) Community stakeholders (*n* = 112) Professional stakeholders (*n* = 63)
PTB – Review Articles (*n* = 13^b^)	2000–2009 (*n* = 1) 2010–2019 (*n* = 7) >/=2020 (*n* = 5)	Europe (*n* = 6) North America (*n* = 4) Oceania (*n* = 2) Africa (*n* = 1)	Qualitative systematic review (*n* = 2) Systematic review (*n* = 5) Integrative review (*n* = 3) Scoping review (*n* = 2) Literature review (*n* = 1)	N/A	Total number of qualitative/mixed methods articles synthesized (*n* = 102)

*FGR* fetal growth Restriction, *GDM* gestational diabetes mellitus, *HDP* hypertensive disorders of pregnancy, *PTB* preterm birth.

^a^Includes 15 studies with mixed populations (Almli 2020, Ferrari 2022, Hoedjes 2011, Hoedjes 2012, Jenkinson 2025, Mattocks 2025, Meaney 2016, Murray-Davis 2022, Nagraj 2019, Nagraj 2023, Ogunwole 2023, Sandsaeter 2019, Smoorenburg 2023, Stanhope 2022, Thomas 2004).

^b^Includes 1 study with mixed populations (Ghisi 2024).

^c^Includes 22 studies with mixed populations (Almli 2020, Bendix 2024, Curran 2017, Ekpenyong 2022, Ferrari 2022, Harrison 2003, Hoedjes 2011, Hoedjes 2012, Jenkinson 2025, Markovic 2006, Mattocks 2025, McCain 1994, Meaney 2016, Murray-Davis 2022, Nagraj 2019, Nagraj 2023, Ogunwole 2023, Sandsaeter 2019, Smoorenburg 2023, Stanhope 2022, Sullivan 2024, Thomas 2004).

^d^Includes 6 studies with mixed populations (Bendix 2024, Bridgeman-Bunyoli 2021, Hoedjes 2011, Hoedjes 2012, Markovic 2006, Sullivan 2024).

^e^Includes 6 studies with mixed populations (Bendix 2024, Bridgeman-Bunyoli 2021, Curran 2017, Ekpenyong 2022, Ferrari 2022, Harrison 2003, Markovic 2006, McCain 1994).

^f^Excludes 5 studies where total participants not reported (Cohen 1999, Moore 2024, Oza-Frank 2018, Timm 2022, van Zijl 2024).

**Fig. 2:**
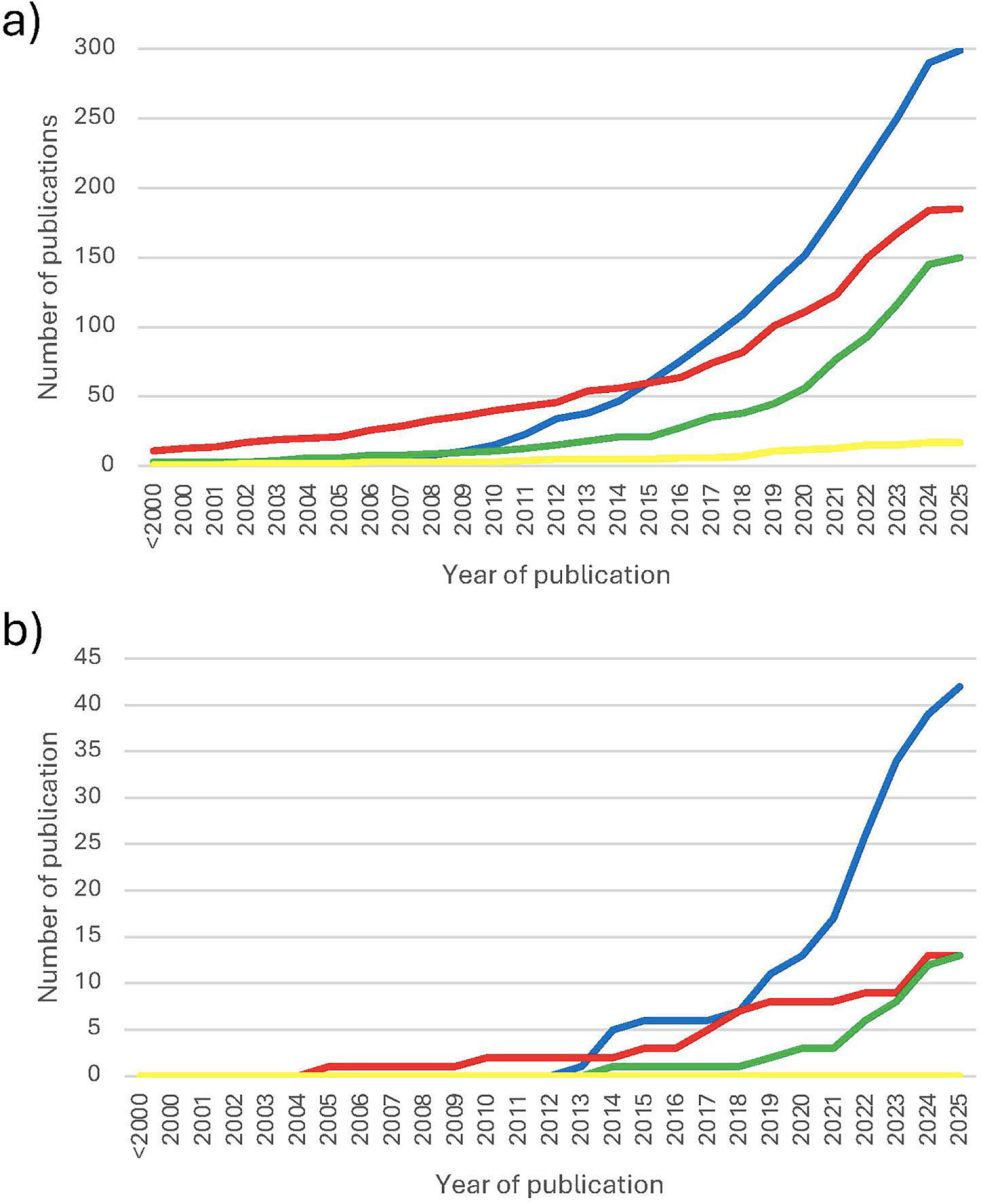
Number of publications on women’s experiences of cardiometabolic pregnancy complications over time. These figures show the number of original and review publications published over time. **a** Number of original publications over time. **b** Number of review publications over time. Legend: Blue – gestational diabetes mellitus. Red – preterm birth. Green – hypertensive disorders of pregnancy. Yellow – fetal growth restriction.

**Fig. 3:**
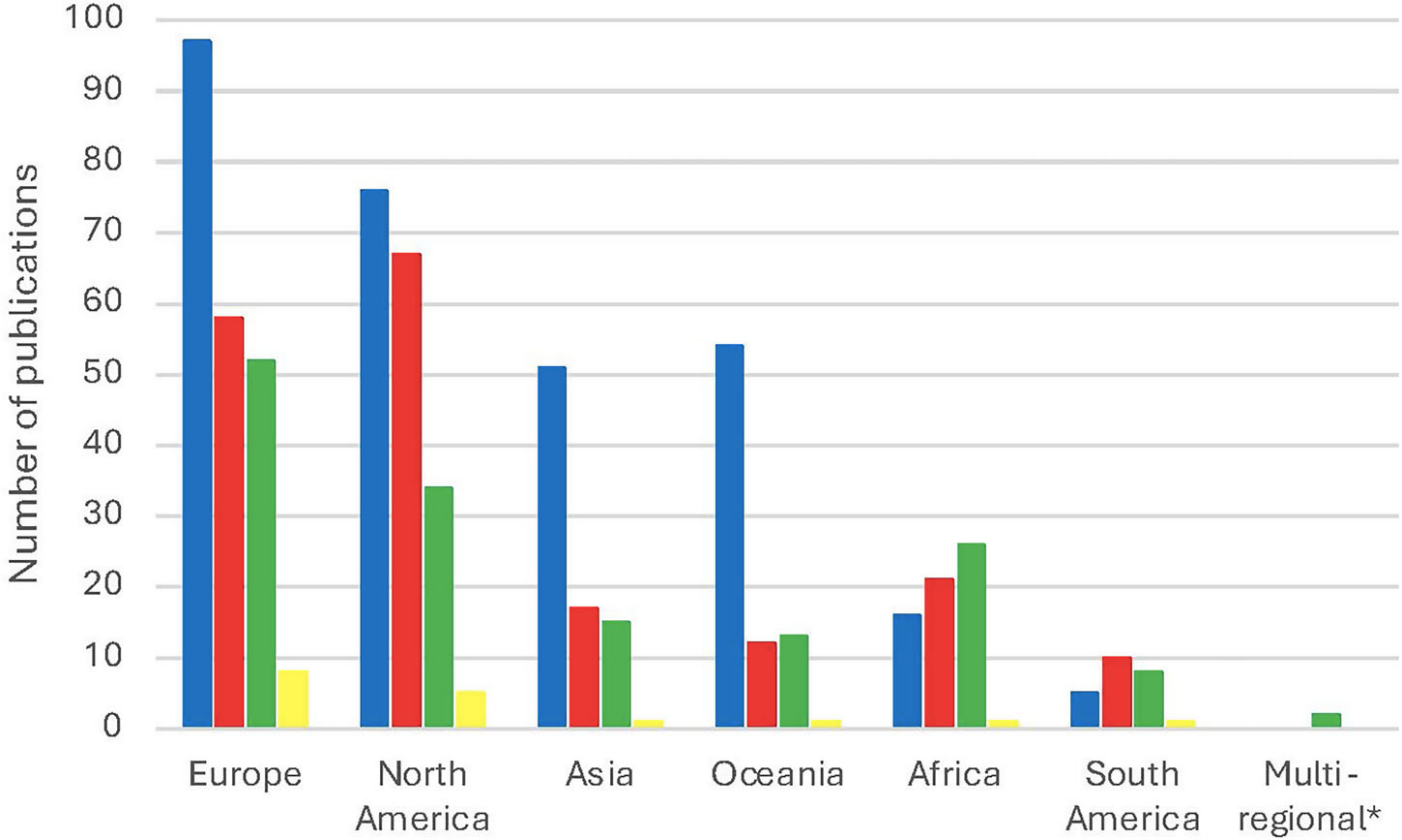
Number of original publications on women’s experiences of cardiometabolic pregnancy complications by region. This figure shows the number of original publications by region of publication. Legend: Blue – gestational diabetes mellitus. Red – preterm birth. Green – hypertensive disorders of pregnancy. Yellow – fetal growth restriction. *Multi-regional publications included one study conducted in Zambia and India and one study conducted in Ethiopia, Haiti and Zimbabwe.

Overall, 623 original research articles exploring women’s experiences of GDM (*n* = 284, 45.6%), HDP (*n* = 128, 20.5%), FGR (*n* = 11, 1.8%), PTB (*n* = 177, 28.4%) and mixed populations (*n* = 23, 3.7%) were identified (see Supplementary Data 1 for article details). Original studies have been published in increasing frequency (Fig. 2), i.e. <2000 (*n* = 15, 2.4%), between 2000 and 2009 (*n* = 40, 6.4%), between 2010 and 2019 (*n* = 220, 35.3%) and >/=2020 (*n* = 348, 55.9%). Most original studies were conducted in Europe (*n* = 204, 32.7%) and North America (*n* = 171, 27.4%), while remaining studies were conducted in Asia (*n* = 82, 13.2%), Oceania (*n* = 77, 12.4%), Africa (*n* = 63, 10.1%) and South America (*n* = 24, 3.9%), with two multiregional studies on HDP, one conducted in Zambia and India and one in Ethiopia, Haiti and Zimbabwe (*n* = 2, 0.3%) (Fig. 3). Data collection methods included interviews (*n* = 407, 65.3%), focus groups (*n* = 47, 7.5%), surveys (*n* = 21, 3.4%), social media content analysis (*n* = 2, 0.3%), case studies (*n* = 2, 0.3%), meetings (*n* = 1, 0.2%) and mixed methods (*n* = 143, 23.0%). Original research explored experiences during pregnancy (*n* = 249, 40.0%), the postpartum period (*n* = 320, 51.4%), both preconception and pregnancy (*n* = 1, 0.2%) and both pregnancy and postpartum (*n* = 53, 8.5%). Research participants included women (*n* = 19941), healthcare professionals (*n* = 3833), partners (*n* = 908) and professional (*n* = 273) and community stakeholders (*n* = 222). Professional stakeholders included researchers, public health specialists, key informants, policy makers, representatives of non-government organizations, non-clinical and/or administrative staff, while community stakeholders included other family members, caretakers, volunteers, community leaders/elders and/or patient advocates.

Overall, 66 review articles exploring women’s experiences of GDM (*n* = 41, 62.1%), HDP (*n* = 12, 18.2%) and PTB (*n* = 12, 18.2%) were identified, with one review exploring GDM, HDP and PTB in combination (*n* = 1, 1.5%). No review articles were identified exploring women’s experiences of FGR. Review articles were primarily published between 2010 and 2019 (*n* = 20, 30.3%) or >/=2020 (*n* = 45, 68.2%), with one study published between 2000 and 2009 (*n* = 1, 1.5%). Reviews were conducted in Europe (*n* = 25, 37.9%), Oceania (*n* = 17, 25.8%), Asia (*n* = 10, 15.2%), North America (*n* = 8, 12.1%), Africa (*n* = 4, 6.1%) and South America (*n* = 2, 3.0%). Study designs included qualitative systematic review (*n* = 25, 37.9%), systematic review (*n* = 23, 34.8%), scoping review (*n* = 11, 16.7%), integrative review (*n* = 4, 6.1%), literature review (*n* = 1, 1.5%), integrative review (*n* = 1, 1.5%) and umbrella review (*n* = 1, 1.5%).

The primary aim of each research article was coded according to an adapted version of the IOM Continuum of Care Model (Table 2)^23^. Articles exploring women’s experiences of GDM (*n* = 342) comprised 49.6% of total identified studies, and primarily focused on treatment and management of GDM during pregnancy (*n* = 119, 34.8%) and optimizing maternal health postpartum (*n* = 74, 21.6%). Research investigating experiences of HDP (*n* = 163) comprised 23.7% of total identified studies and focused on treatment and management during pregnancy (*n* = 43, 26.4%) and healthcare systems and delivery (*n* = 33, 20.2%), along with optimizing and maintaining maternal health postpartum (*n* = 27, 16.6%) and psychosocial health during pregnancy (*n* = 23, 14.1%). Articles exploring experiences of FGR (*n* = 17) formed only 2.5% of identified studies and focused on treatment and management during pregnancy (*n* = 7, 41.2%). Research exploring PTB (*n* = 198) comprised 28.7% of identified studies and focused on postpartum experiences, with prominent areas of investigation including psychosocial health (*n* = 67, 33.8%) and breastfeeding (*n* = 37, 18.7%). Experiences of treatment and management of increased risk of preterm birth during pregnancy (*n* = 24, 12.1%) were also commonly investigated.

**Table 2:** Categorization of original research articles based on study aim or primary area of investigation

Category	Primary Area of Investigation (Definition)	GDM (*n* = 342^a^)	HDP (*n* = 163^b^)	FGR (*n* = 17^c^)	PTB (*n* = 198 ^d^)
*Prevention (Pregnancy)*					
Prevention	Articles primarily explore women’s experiences of medical, lifestyle (diet/activity) and/or pharmacotherapy interventions or behaviours to prevent the onset of cardiometabolic conditions during pregnancy.	3.8% (*n* = 13)	3.7% (*n* = 6)	0% (*n* = 0)	8.1% (*n* = 16)
*Treatment (Pregnancy)*					
Screening and Diagnosis	Articles primarily explore women’s experiences of screening or diagnosis during pregnancy. This includes women’s understanding and perceived risk of the cardiometabolic pregnancy complication.	4.4% (*n* = 15)	8.6% (*n* = 14)	0% (*n* = 0)	6.6% (*n* = 13)
Treatment and Management	Articles primarily explore women’s experiences of medical, lifestyle (diet/activity) and/or pharmacotherapy interventions or behaviours during pregnancy. This includes medical consults, such as antenatal consults for preterm labor.	34.8% (*n* = 119)	26.4% (*n* = 43)	41.2% (*n* = 7)	12.1% (*n* = 24)
Psychosocial Health	Articles primarily explore psychological, emotional and/or social aspects of women’s health during pregnancy.	11.1% (*n* = 38)	14.1% (*n* = 23)	17.6% (*n* = 3)	9.1% (*n* = 18)
Healthcare Systems and Delivery	Articles primarily relate to functioning of the healthcare system and explore women’s experiences of quality, distribution or access to resources during pregnancy.	14.6% (*n* = 50)	20.2% (*n* = 33)	17.6% (*n* = 3)	8.6% (*n* = 17)
*Maintenance (Postpartum)*					
Screening for Cardiometabolic Conditions	Articles primarily explore women’s experiences of screening for a chronic disease after pregnancy. This includes women’s understanding and perceived risks of the condition.	10.2% (*n* = 35)	6.7% (*n* = 11)	0% (*n* = 0)	0.5% (*n* = 1)
Optimizing Maternal Health	Articles primarily explore women’s experiences of medical, healthy lifestyles (diet/activity) and/or pharmacotherapy interventions or behaviours to optimize general or cardiometabolic health, including in relation to cardiometabolic complications in the postpartum period.	21.6% (*n* = 74)	16.6% (*n* = 27)	11.8% (*n* = 2)	3.5% (*n* = 7)
Breastfeeding	Articles primarily explore women’s experiences of breastfeeding.	2.0% (*n* = 7)	0% (*n* = 0)	5.9% (*n* = 1)	18.7% (*n* = 37)
Healthcare Systems and Delivery	Articles primarily relate to the functioning of the healthcare system and explore women’s experiences of quality, distribution or access to resources in the postpartum.	4.4% (*n* = 15)	4.9% (*n* = 8)	0% (*n* = 0)	4.5% (*n* = 9)
Psychosocial Health	Articles primarily explore psychological, emotional and/or social aspects of women’s health in the postpartum, including women’s experiences of parenting after a cardiometabolic complication of pregnancy.	2.0% (*n* = 7)	6.1% (*n* = 10)	17.6% (*n* = 3)	33.8% (*n* = 67)

^a^32 articles were coded with multiple primary aims.

^b^12 articles were coded with multiple primary aims.

^c^2 articles were coded with multiple primary aims.

^d^11 articles were coded with multiple primary aims.

### Summary of content analysis of abstracts using Leximancer

The Leximancer generated figures for GDM, HDP, PTB and FGR are presented below. The visual displays are heat-mapped for most prevalent themes, with the red bubble denoting the most prevalent theme, followed by yellow, green, blue and purple. Within each theme, the words represent the meaningful concepts that inform the theme, with the lines showing the path most likely travelled between concepts, representing the proximity of those concepts within the text. More data input informs the software, which is then capable of generating more densely populated visual displays with more meaningful connections between concepts. Across GDM, HDP and FGR, the most prevalent theme was *‘Women’*, whereas the most prevalent theme in PTB was *‘Mothers’*, highlighting a shift of perspective in the abstracts from women’s experiences of their cardiometabolic complication of pregnancy, to the experience of being a mother to a preterm baby. Only the HDP visual display identifies CVD as a key concept, informing the theme of *‘Women’*. While some themes across the four conditions related are specifically to postpartum health, such as ‘*Diabetes’* (T2DM) in GDM and *‘Milk’* (Breastfeeding) in PTB, no theme is specifically related to future cardiovascular risk.

### Investigating women’s experiences of gestational diabetes

**Fig. 4:**
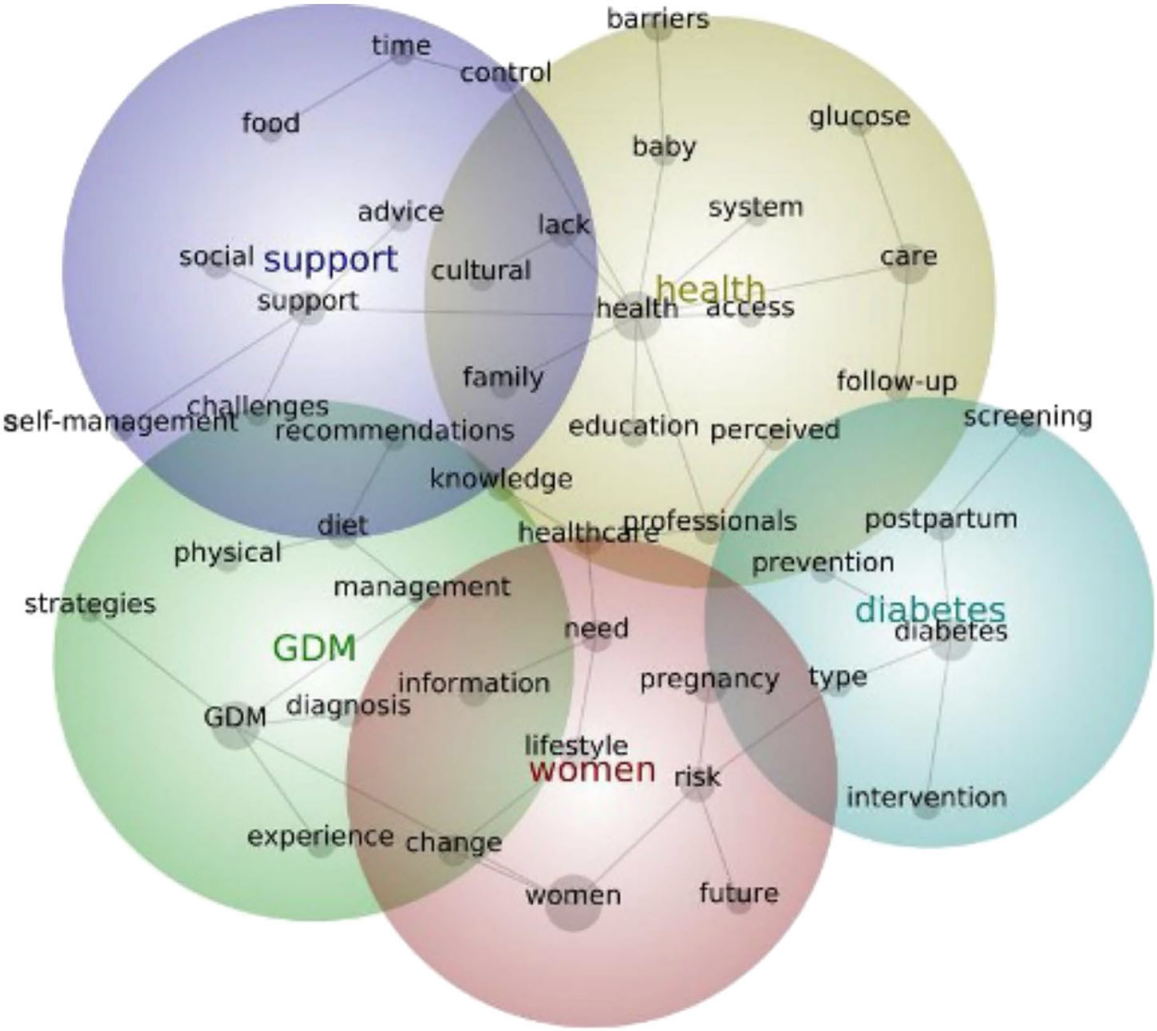
Leximancer-generated concept map of prevalent themes within GDM literature. This figure shows the Leximancer-generated concept map of prevalent themes within GDM literature. In order of prevalence: red, yellow, green, blue, purple. GDM gestational diabetes mellitus.

Key themes generated from the content analysis of GDM abstracts using Leximancer^24^, in order of prevalence, relate to 1) *Women*, 2) *Health*, 3) *GDM*, 4) *Diabetes*, 5) *Support* (Fig. 4). *‘Women’* comprised of concepts including risk, lifestyle, pregnancy, change, need, information, healthcare and future, suggesting that research reported on women’s experiences of healthcare, including information on risk, lifestyle changes and future implications. ‘*Health*’ related to concepts including care, professionals, barriers, lack, education, access and follow-up, suggesting that women’s health was dependent on interactions with professionals, experiencing lack of support and education or barriers to accessing care or follow-up. ‘*GDM*’ comprised of concepts including diet, management, diagnosis and knowledge, suggesting that women discussed experiences of their GDM diagnosis, diet recommendations, and other management strategies including physical exercise. *‘Diabetes*’ comprised of concepts including postpartum, intervention, type, screening and prevention, suggesting that women discussed the risk of type 2 diabetes, including prevention and screening. The separation between *‘GDM’* and *‘Diabetes’* highlights that there is research surrounding women’s experiences of GDM management and experiences of T2DM risk in the postpartum as two distinct themes. *‘Support*’ comprised of concepts such as social, cultural, advice, recommendations, self-management and food.

### Investigating hypertensive disorders of pregnancy

**Fig. 5:**
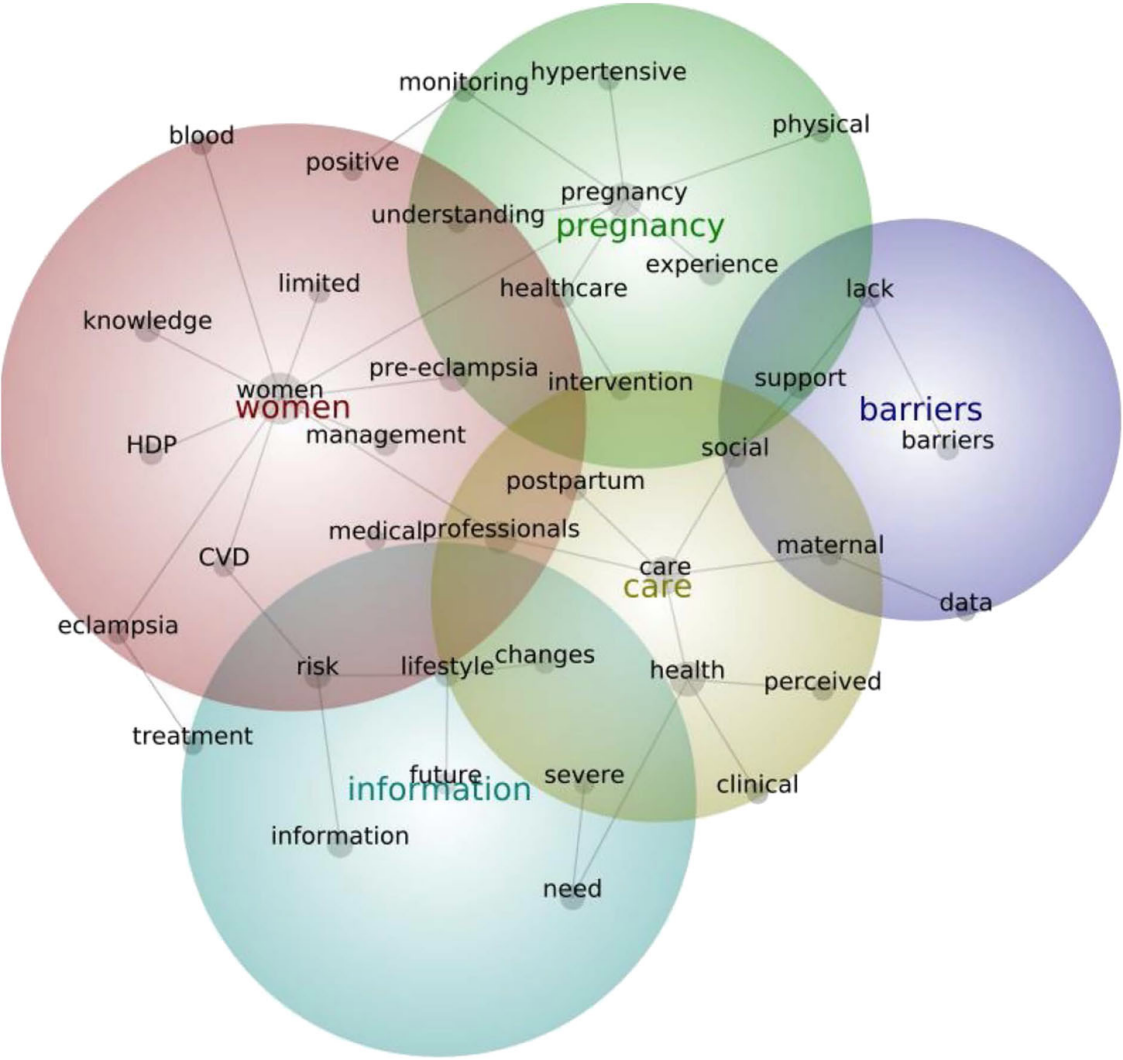
Leximancer-generated concept map of prevalent themes within HDP literature. This figure shows the Leximancer-generated concept map of prevalent themes within HDP literature. In order of prevalence: red, yellow, green, blue, purple. CVD cardiovascular disease, HDP hypertensive disorders of pregnancy.

Key themes generated from the analysis of HDP abstracts, in order of prevalence, relate to 1) *Women*, 2) *Care*, 3) *Pregnancy*, 4) *Information*, 5) *Barriers* (Fig. 5). *‘Women’* comprised of concepts including knowledge, management, medical professionals, and CVD, highlighting women’s experiences of the medical management of HDP, including their understanding of pre-eclampsia and eclampsia, symptoms, and risk of future CVD. *‘Care’* comprised of concepts including maternal, health, postpartum, and social, focusing on women’s experiences of their healthcare, with studies reporting on feelings of dissatisfaction with care provided, the need for social and psychological support in the postpartum, alongside recommendations for improving care. *‘Pregnancy’* comprised of themes such as hypertensive, monitoring, physical, experience, highlighting the experience of a hypertensive pregnancy, monitoring, and any physical symptoms. *‘Information’* comprised of themes such as future, risk, lifestyle and severe, suggesting that women wanted ongoing, consistent information about the severity of disease, as well as ongoing future risk and any suggested lifestyle changes. *‘Barriers’* included concepts like support, lack and data, exploring obstacles to healthcare provision and women’s experiences of support (or lack thereof), and data related to maternal blood pressure or other parameters that might inform management of hypertensive disorders of pregnancy.

### Investigating preterm birth

**Fig. 6:**
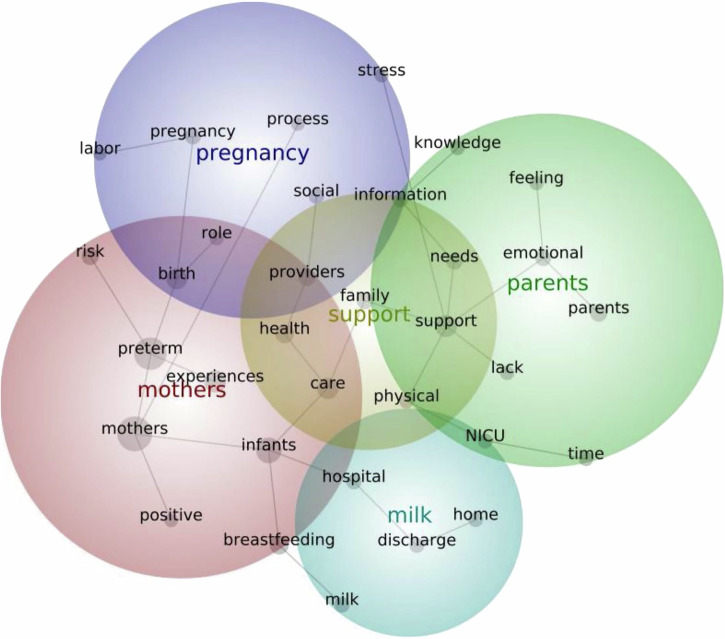
Leximancer-generated concept map of prevalent themes within PTB literature. This figure shows the Leximancer-generated concept map of prevalent themes within PTB literature. In order of prevalence: red, yellow, green, blue, purple. NICU neonatal intensive care unit.

Key themes generated from the content analysis of PTB abstracts, in order of prevalence, relate to 1) *Mothers*, 2) *Support*, 3) *Parents*, 4) *Milk*, 5) *Pregnancy* (Fig. 6). *‘Mothers*’ relates concepts such as preterm, infants, experiences, birth and breastfeeding, suggesting that women’s key experiences revolved about that of becoming a mother, focusing on the birthing experience and caring for and breastfeeding their preterm infant. *‘Support’* included concepts like care, health, providers, family, needs and information, suggesting that women sought support from their healthcare providers and family and needed appropriate care and information in their premature birth journey. ‘*Parents*’ included themes such as emotional, knowledge, lack, time and feeling, which supports the primary theme of motherhood with the emotions and feelings of becoming a parent, as well as highlighting experiences such as desire for or lack of knowledge and time. *‘Milk’* related to hospital, discharge, home and NICU, exploring women’s experiences of their milk production and breastfeeding from birth in hospital through to discharge to the home environment. *‘Pregnancy’* related to concepts such as social, role, stress, process and labour, relating to women’s risk of preterm birth, including experienced stress and social stigma or the shock of an unexpected early labour and the women’s perceived role in the process.

### Investigating fetal growth restriction

**Fig. 7:**
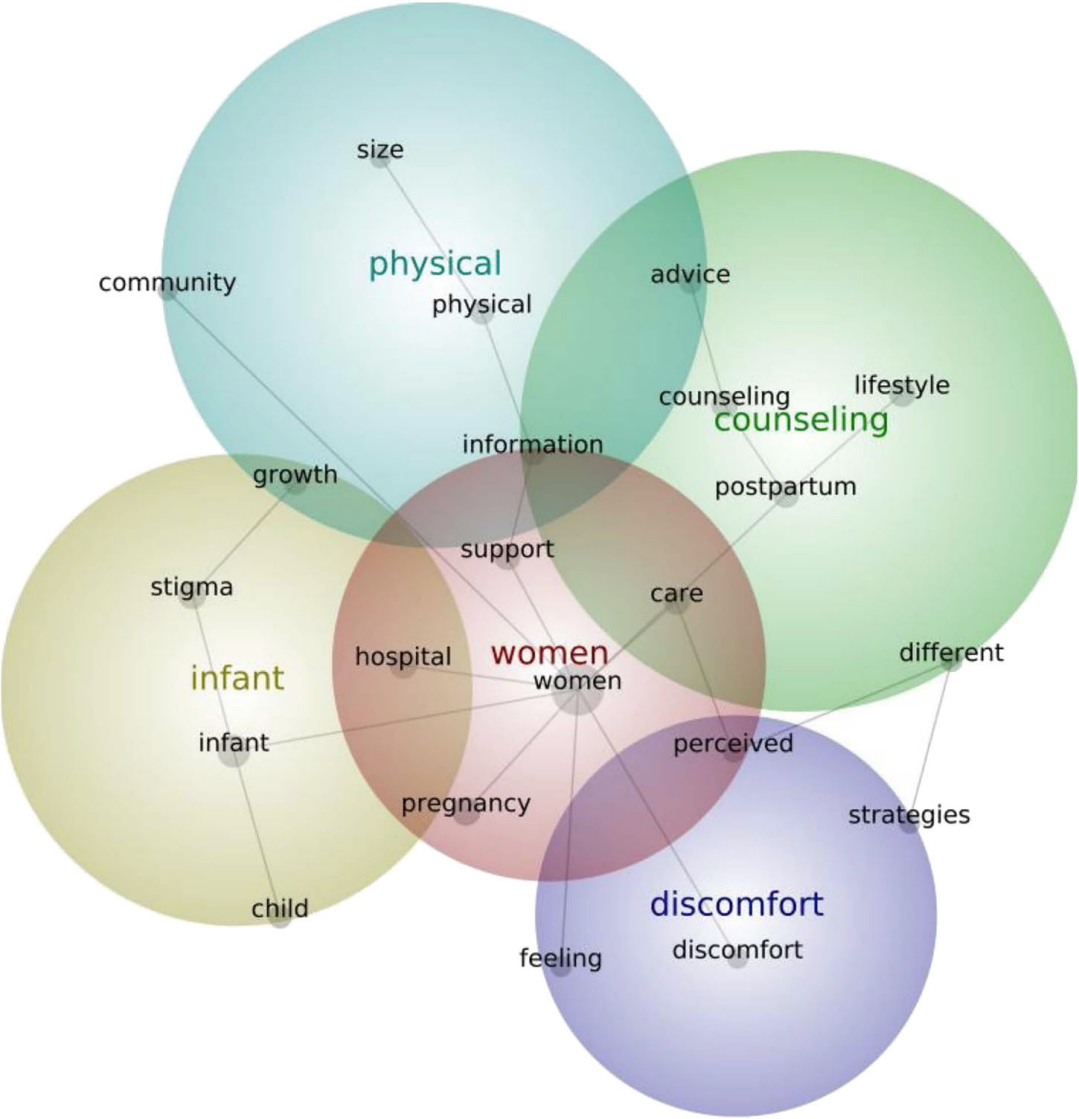
Leximancer-generated concept map of prevalent themes within FGR literature. This figure shows the Leximancer-generated concept map of prevalent themes within PTB literature. In order of prevalence: red, yellow, green, blue, purple.

Key themes generated from the analysis of FGR abstracts, in order of prevalence, relate to 1) *Women*, 2) *Infant*, 3) *Counselling*, 4) *Physical*, 5) *Discomfort* (Fig. 7). *‘Women’* included concepts such as support, care, hospital and pregnancy, examining how women with FGR perceive their care and interactions within the hospital, highlighting experiences of inadequate support and their desire for detailed information during pregnancy. *‘Infant’* included concepts such as growth and stigma, exploring women’s experiences of their infant’s health, particularly anxiety surrounding feeding and growth, with some women experiencing limited community support and stigma for having a small baby. *‘Counselling’* included concepts such as lifestyle, postpartum and advice, addressing women’s experiences of the advice they received during pregnancy and the postpartum period, with some women seeking counselling on maintaining a healthy lifestyle in the postpartum period. *‘Physical’* included concepts such as size, information and community, exploring women’s concerns about their baby’s size and growth, as well as related to the infant theme, with perceptions of how their baby may be perceived in their community. *‘Discomfort’* included concepts such as feeling, capturing women’s feelings of anxiety and discomfort related to the additional appointments and monitoring.

## Discussion

This scoping review highlights the breadth of research exploring women’s experiences of cardiometabolic complications of pregnancy. This approach provides high level insights, with GDM being the area with the most prolific research, followed by PTB and HDP, with FGR less well represented (see Fig. 2). Based on our modified model of the IOM continuum of care, the most represented stage of care in the original literature was the postpartum period (51.4%), followed by the pregnancy period (40.0%). 8.5% of studies comprised of a mixed population of women either during pregnancy or in the postpartum period, and only one study (0.2%) comprised of a mixed population of women in the pre-conception period or during pregnancy. The one paper exploring experiences during pre-conception and pregnancy included women recruited from a specialist pre-pregnancy planning clinic out of a population of women with type 1 diabetes^25^, discussing their risk of pre-eclampsia. This is a population which is likely much more experienced in managing health conditions and cardiovascular risk than the general population of women of childbearing age.

Psychosocial health was a strong focus of both FGR and PTB literature, with the majority of PTB papers having psychosocial health in the postpartum as the primary area of investigation. Psychosocial health was also a key concept prevalent in the Leximancer analysis of FGR and PTB literature. In GDM, HDP and FGR literature, treatment and management during pregnancy was the leading primary area of investigation, followed by optimizing maternal health in the postpartum in women with GDM, and experiences of healthcare systems and delivery during pregnancy in women with HDP. The focus on women’s experiences of healthcare systems and delivery may relate in part to the higher proportion of literature on HDP originating in low- and middle-income countries in Africa (see Fig. 3) with developing healthcare infrastructure, with the medical aspects of management emphasized in HDP papers. Interestingly, cardiovascular disease was only identified as a key concept by Leximancer for the HDP papers.

Overall, there is a comparative sub-representation of South America in the literature identified in this review across each condition (see Fig. 3), highlighting an area in which more research is warranted to inform country or region-specific screening and preventative maternity medicine. We also acknowledge that this may be influenced by our search criteria limited to studies in English or where an English translation is readily available.

This review demonstrates the potential for qualitative research in women’s healthcare and primary T2DM and CVD prevention. The increasing rate of publication highlights the opportunity to use this literature to inform woman-centred care. This is particularly evident in GDM and HDP literature, with a large proportion of all original articles published since 2020 (56% GDM, 70% HDP). Qualitative research may inform researchers, providers and policy makers on women’s preferences for care, and practical strategies such as when and how best to engage women and increase uptake of preventative healthcare against T2DM and CVD, which can be notoriously challenging^26,27^. There is also the opportunity to pair qualitative research with implementation science principles and frameworks to translate findings into tangible interventions and outcomes^28,29^.

The findings of this review highlight unique differences in experiences between populations of women, suggesting that women with certain cardiometabolic complications may warrant individualized approaches in relation to CVD prevention strategies including counselling, screening and interventions^30^. Consistent with our findings, others have noted that while lifestyle interventions may reduce incidence of T2DM after GDM, less is known about women with other complications of pregnancy^30^. This reflects our findings that lifestyle and associated challenges were a greater focus of GDM literature. Qualitative research in GDM appeared more medicalized than other fields, with roughly one-fifth of the sample size in GDM being healthcare professionals. This may contribute to the proportion of GDM papers addressing awareness of increased risk of T2DM and future cardiometabolic complications, with a focus on pre- and postpartum screening, management and lifestyle change. HDP research also involved a higher proportion of healthcare professionals discussing women’s experiences, often related to screening and monitoring of a hypertensive pregnancy, with a focus on maternal health and intrapartum medical management. In addition, more HDP studies were published in developing countries. Many of these papers highlighted low health literacy, increased misconceptions about HDP and poor understandings of the link between HDP and future CVD, as well as geographically and culturally specific challenges in follow-up.

Comparatively to GDM and HDP, PTB and FGR papers focused on women’s psychosocial outcomes, as well as the experience of parenting a small or preterm infant, with less of a focus on maternal cardiometabolic health. Less than one-tenth of the sample size in PTB literature and FGR literature was healthcare professionals, with partners and community stakeholders were more represented in the PTB literature. This may represent how partners may feel more involved in the experience of having a preterm birth as the other ‘parent’, or the psychosocial aspects of having a medically complex child within a family unit, rather than a discussion or understanding of maternal health implications in relation to CVD. This likely contributes to the fact that postpartum screening for cardiometabolic conditions was not a key category of research across these conditions.

With the Leximancer analysis, while T2DM was identified as a key theme in GDM research, heart health was not identified as a primary theme across any condition, and CVD was only identified as a key concept for HDP, highlighting the potential lack of awareness or lack of priority of future cardiovascular wellbeing among women. Commonalities across each field include commentary on psychosocial stress and mental health and the emphasis on social support and engaging family with care.

The implications of this research from pre-conception through to postpartum are important to consider for clinicians across healthcare domains, including primary care, obstetrics, and cardiology. Reinforcing the understanding that these pregnancy complications represent an early positive cardiovascular ‘stress-test’ will require a common understanding by clinicians to ensure consistent messaging. It is important to discuss preventative healthcare and screening early, given the high psychosocial stress a woman may experience after delivery, especially if women experience an early delivery or unexpected interventions. This messaging is then crucial to reinforce in the postpartum. Using the concept of a ‘fourth trimester’^31^ may be useful for clinicians, to consider the postpartum as a critical window of opportunity to optimize women’s cardiovascular health. Acknowledging that maternal postpartum care is generally underutilized by women post-delivery^32^, and that women experiencing FGR and PTB will likely prioritize the care of their infant over their own health, it is crucial that there are clear referral pathways and criteria for these women, and effective utilization of available resources to providers and health services (e.g. combined cardio-obstetric clinics). It is pertinent for cardiology teams when these women do engage with specialist care (at any time) to take a routine history of cardiometabolic complications of pregnancy, and to understand that there may be potential barriers to engagement or follow up with care such as birth trauma and experiences of stigma, with potential adaptations such as flexible scheduling, telehealth and/or considerations of the accessibility of any investigations or interventions.

This scoping review identifies opportunities for future research priorities. The relative paucity of studies relating to maternal experiences of FGR is a key domain to be explored, acknowledging that identifying truly growth restricted infants can be challenging^33^. Spontaneous PTB and FGR, while independently risk factors for poor cardiometabolic health outcomes, may coincide with classical risk factors for T2DM and CVD (smoking or other pregnancy complications including GDM and HDP) favouring the recommendation to study these in tandem^34^. The well-developed body of literature around experiences of having a preterm infant may be synthesized further in review papers to identify appropriate opportunities for healthcare intervention. The lack of research into lifestyle change to optimize cardiometabolic health amongst mothers with FGR and spontaneous PTB highlights potential for further study. Qualitative evidence across all conditions may also be used to inform timing of interventions, identifying points of care during a period in which women are generally already engaging with health services at which they may be receptive to advice on future CVD and T2DM risk reduction, including preventative screening and lifestyle counselling^35^. In particular, we note a relative paucity in literature exploring longitudinal experiences of women across preconception, pregnancy and the postpartum. It is vital to engage women in their understanding of cardiometabolic risk factors from as early as preconception and explore how the management of risk factors may change from pregnancy to postpartum, to comprehensively tailor screening and preventative initiatives. Synthesizing women’s perspectives across the timespan in which they may engage with primary, or specialist obstetric care can guide researchers in identifying sensitive and appropriate opportunities to implement follow up for future maternal health and wellbeing.

The key strength of this study is the high-level overview provided of qualitative research encompassing the perspectives of women, healthcare providers and stakeholders. There is increasing recognition that cardiometabolic dysregulation evident following GDM, HDP, spontaneous PTB and FGR has implications for future cardiovascular health^36–39^. Women tend to have highly accurate recall of pregnancy-related events^40^, and their experiences should be used by primary health care providers to inform T2DM and CVD primary prevention^41^. To our knowledge, this is the first review to compare qualitative research both across multiple cardiometabolic conditions and across the continuum of care from pregnancy to postpartum. This paper also uniquely adapts the IOM Continuum of Care Model to analyse trends of qualitative experiences of at-risk women across pregnancy and the postpartum as well as adopting novel methods of qualitative data interpretation by using Leximancer. These insights can be used to inform healthcare that acknowledges prior experiences and highlights opportunities for intervention^23^. This review comprehensively identifies priorities for future research, whilst highlighting the scope of existing extensive research to prevent research wastage^42^. In particular, our findings clearly demonstrate the increasing rate of qualitative publications, highlighting ample material to guide implementation research and clinical care.

A unique feature of this review was the use of Leximancer software, a machine-learning, data-mining tool, to analyse the key findings of each eligible study as reported in the study abstract^43^. Leximancer enables systematic and relational analysis of key concepts, mitigating challenges with inconsistent and often anecdotal interpretation of large volumes of data^44^. Leximancer enables rapid interpretation of large volumes of text and facilitated a high-level comparison and interpretation of key findings from over 600 studies exploring women’s experiences of a cardiometabolic pregnancy complication. Clinicians could refer to these concepts when considering what to investigate or be cognisant of when consulting with women who have experienced a cardiometabolic pregnancy complication. However, while Leximancer is useful in assessing concepts in vast textual data, it cannot provide nuanced information. As this study is a scoping review, we only intended to scope and summarise the existing literature. The results from abstracts of individual studies were provided to Leximancer and an overview is captured without synthesis of each woman’s unique perspective. This is a key limitation inherent to our study. The Leximancer findings do not generate specific recommendations for clinical practice, nor can they be generalised across specific and often highly individualised contexts. A meta-thematic analysis (which was not possible with this breadth of studies) of women’s perspectives, likely with additional limits such as by country or region, is recommended to generate a nuanced and detailed interpretation of women’s experiences of living with a cardiometabolic pregnancy complication.

This study captures global perspectives of women’s experiences. Although most papers represented European and North American publications, there is also representation across Asia, Oceania, Africa and South America across each condition we examined. However, we did not extract demographic information and only included studies that were readily available in English, which may disproportionately capture perspectives of English-speaking women. There is also limited ability to comment on ethnically diverse experiences or women experiencing socioeconomic disadvantage based on the scope of this review. Future investigation and tailored analysis of these women’s insights to address health disparities and tailored healthcare implementation in ethnically diverse or disadvantaged populations is needed^11,45^.

Not all PTB papers specified whether women experienced spontaneous versus medically induced PTB. We did not exclude these papers as medically induced PTB may be due to other cardiometabolic conditions of interest, including GDM, HDP and FGR, however this limits interpretation of the implications specifically of spontaneous PTB. The breakdown of healthcare practitioners into primary care practitioners, obstetrician/gynaecologists, other specialists and allied health workers was also not extracted. This valuable information can be used to explore how a ‘fourth trimester’ and postpartum monitoring may look like with shared responsibilities between obstetrics, general practice, allied health and other specialists, with appropriate transitions of care and multidisciplinary collaboration^34,46^.

In conclusion, this scoping review highlights the breadth of qualitative literature around cardiometabolic pregnancy complications. The research primarily centred around treatment and management, optimizing postpartum maternal health and healthcare delivery, with Leximancer analysis reflecting the emotional impact and the importance of support and information for women experiencing these high-risk pregnancies. Overall, a focus on future maternal health was represented across all conditions, with the greatest focus on future cardiometabolic risk in GDM, followed by HDP. These findings offer valuable high-level insights for enhancing healthcare interventions for women and highlight priority areas for future research. Findings emphasize the need for further investigation of maternal experiences of FGR and spontaneous PTB and suggest collectively examining strategies to optimize cardiometabolic health in these women. Researchers can refer to the studies included in this this review as a starting point to creating contextualised evidence-based insights to inform future guidelines. Contextual data such as age, region, socio-economic status or cultural and linguistic diversity may be extracted from these studies to help identify sensitive and appropriate opportunities to implement management and follow-up targeting future maternal cardiometabolic health and wellbeing^32^.

## Methods

A scoping review was undertaken in accordance with the methodological framework outlined by Arksey and O’Malley^47^ and the Preferred Reporting Items for Systematic Reviews and Meta-Analyses extension for Scoping Reviews (PRISMA-ScR) Checklist (Supplementary Table S1)^48^. This review was prospectively registered on Open Science Framework [10.17605/OSF.IO/ZMHEG]^49^.

### Data sources

Seven electronic databases were searched from inception to July/August 2022 (MEDLINE, CINAHL, EMBASE, PsycInfo, Emcare and EBM Reviews). An updated search was performed on the same databases from 2022 to February 2025. Search terms broadly related to *qualitative research* and pregnancy complications including *GDM, HDP, FGR and PTB*. There were no limits placed on year of publication. Given the large number of articles generated, reference lists of included full texts were not hand-searched for additional eligible articles. Supplementary Table S2 details the search strategy.

### Main outcome measures

The primary outcome of this review was to provide a comprehensive overview of the extent, range and nature of qualitative research investigating experiences of women at-risk of or diagnosed with cardiometabolic pregnancy complications.

### Eligibility criteria

The search strategy was informed by the SPIDER (Sample, Phenomenon, Design, Evaluation, Research type) tool for qualitative evidence synthesis^50^. See Supplementary Table S3 for a detailed summary of inclusion and exclusion criteria.

The sample included women at risk of or diagnosed with GDM, HDP, FGR or PTB during pregnancy, and the phenomenon was women’s or stakeholder perspectives of women’s experiences during or following these pregnancies. At-risk populations were accepted if studies described selection of participants based on individual risk rather than general sociodemographic factors. Stakeholders included (but were not limited to) family members, healthcare professionals, policy makers and members of non-governmental organizations. Qualitative or mixed method research studies were included. As per the methodology of a scoping review^51^, there was no formal evaluation of the quality of included articles.

Given the intention to explore maternal experiences for reduction of future cardiometabolic risk, studies pertaining to infant health, including perceptions of healthcare provided to the infant, breastmilk supply and experience of healthcare provided in the neonatal intensive care unit (NICU) or acute clinical settings for infant care were excluded. Studies relating to maternal wellbeing, the psychosocial implications of caring for a preterm or FGR infant or the decision to breastfeed following discharge from an acute clinical setting were included as breastfeeding may contribute to reducing women’s future T2DM and CVD risk^11^. Book sections, theses, film/broadcast, opinion articles, narrative review articles, pre-prints, conference abstracts, abstracts with no full study available and protocol papers were excluded, as were studies not published in English.

### Article screening

Screening was managed using Covidence^52^. Duplicates were removed manually. Two independent reviewers (MX, SL) assessed titles and abstracts against the selection criteria. 10% overlap was used to ensure agreement between decisions. Any conflicts were additionally assessed by a third reviewer (LM) and selection criteria were refined or elaborated accordingly. Four independent reviewers (MX, SL, MW, CK) assessed full-text articles, again with 10% overlap. Conflicts were resolved by discussion.

### Data collection and analysis

Study author, publication year, country, study design, aims, sample population, data collection techniques and analysis approach as well as an overview of key themes were extracted from eligible studies into Microsoft Excel (version 16). Data on total studies published on each cardiometabolic condition, and breakdown of year range, continent, study design, life stage and total participants were synthesized using descriptive statistics. Studies investigating mixed populations or women with varied cardiometabolic conditions were reported separately under each condition investigated. We recognize that not all individuals who experience a cardiometabolic pregnancy complication identify as women. The use of the term women throughout this article is consistent with articles eligible for inclusion, but may reflect the perspectives of any individual who experiences a cardiometabolic pregnancy complication, irrespective of gender^53^.

To explore the breadth of research, a framework to categorize studies was developed a priori. Categories were informed by the IOM Continuum of Care Model^23^, and recommendations/implications from the American Heart Association Scientific Statement on Adverse Pregnancy Outcomes and Cardiovascular Disease Risk^11^. The IOM model was adapted to categorize healthcare as Prevention (of a cardiometabolic pregnancy complication), Treatment (of the cardiometabolic complication during pregnancy) and Maintenance (of general health and prevention of cardiometabolic disease postpartum) (Supplementary Fig. S1). This model was chosen to explore the breadth of research investigating women’s experiences across the continuum of healthcare. Universal prevention was excluded given our population of at-risk women. Additional categories were iteratively developed as required. Rationale for the creation of each category is outlined in Supplementary Table S4. Data were synthesized in Microsoft Excel using descriptive statistics.

### Concept analysis using Leximancer

Concept analysis was conducted using Leximancer^24^. Study findings as outlined in the study abstract, were uploaded into Leximancer as they concisely summarized important findings of each study and key issues shaping women’s experiences. Leximancer co-conducts semantic and relational analysis of textual data^43^. Semantic analysis involves identifying semantically related words (concepts) to develop a categorical dictionary. Relational analysis involves the application of the categorical dictionary to the text. Statistical, data mining and network analyses are performed on the categorized text, with concept frequency, total concept connectedness, direct inter-concept relative co-occurrence frequency and total inter-concept co-occurrence used to inform the visual depiction of the concept map and generation of key themes^43^. The visual display is heat mapped, with red representing the most prevalent them, followed by yellow, blue, purple, then green representing the least prevalent theme. The circle size does not reflect the theme’s importance. Default settings were used to analyse data and generate visual displays, with variations to default settings outlined (Supplementary Table S5). The theme size of the visual display was adjusted to reflect the five most common themes. Two authors (SL, WX) independently interpreted the visual display, reviewed content from abstracts that directly informed each theme and discussed the relevance of each theme until a consensus was reached.

## Supplementary information

Supplementary information

Supplementary data 1

## Data Availability

All data generated or analysed during this study are included in this published article and its supplementary data file.
