# Supplementary material for: A scoping review exploring women’s experiences of cardiometabolic pregnancy complications and future cardiovascular health implications
Source: NPJ Cardiovasc Health. 2026 Mar 23;3:11. doi: 10.1038/s44325-026-00107-8 (PMC13009497; doi:10.1038/s44325-026-00107-8)
Supplement: Supplementary file 1 — Supplementary information [file 44325_2026_107_MOESM1_ESM.pdf]

A scoping review exploring women's experiences of cardiometabolic pregnancy complications and future cardiovascular health implications

---

Supplementary Information

Table of Contents

Figure S1: Diagrammatic representation of categorisation of studies according to the primary aim (*inspired by the IOM Continuum of Care[1]*)....2

Table S1: Preferred Reporting Items for Systematic reviews and Meta-Analyses extension for Scoping Reviews (PRISMA-ScR) checklist. 3

Table S2: Search strategy. 5

Ovid MEDLINE (R)....5

CINAHL Plus 6

EMBASE Classic + EMBASE....7

APA PsycInfo....9

Ovid Emcare 9

All EBM Reviews – Cochrane DSR, ACP Journal Club, DARE, CCA, CCTR, CMR, HTA, and NHSEED11

Table S3: Inclusion and exclusion criteria. 13

Table S4: Rationale for categorisation of studies....13

Table S5: Summary of Leximancer analysis settings (variations to default settings) used to generate concept maps....16

References....17

Figure S1: Diagrammatic representation of categorisation of studies according to the primary aim (*inspired by the IOM Continuum of Care*[1]).

This figure demonstrates the categorisation of studies according to primary aim using an adapted model of the IOM continuum of care. Abbreviations: CVD - cardiovascular disease. IOM – Institute of Medicine. T2DM – type 2 diabetes mellitus.

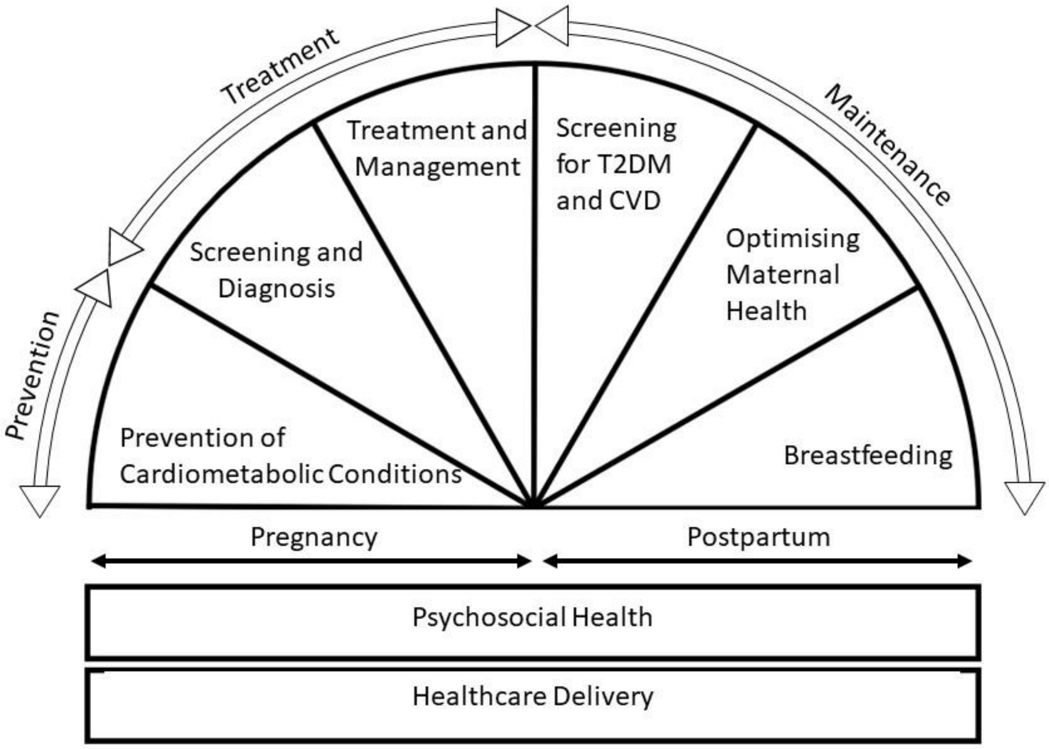

Table S1: Preferred Reporting Items for Systematic reviews and Meta-Analyses extension for Scoping Reviews (PRISMA-ScR) checklist.

This table demonstrates the PRISMA-ScR checklist for this scoping review.

SECTION	ITEM	PRISMA-ScR CHECKLIST ITEM	REPORTED ON PAGE #
**TITLE**			
Title	1	Identify the report as a scoping review.	pg.1
**ABSTRACT**			
Structured summary	2	Provide a structured summary that includes (as applicable): background, objectives, eligibility criteria, sources of evidence, charting methods, results, and conclusions that relate to the review questions and objectives.	pg.2
**INTRODUCTION**			
Rationale	3	Describe the rationale for the review in the context of what is already known. Explain why the review questions/objectives lend themselves to a scoping review approach.	pg.3-4
Objectives	4	Provide an explicit statement of the questions and objectives being addressed with reference to their key elements (e.g., population or participants, concepts, and context) or other relevant key elements used to conceptualize the review questions and/or objectives.	pg.4
**METHODS**			
Protocol and registration	5	Indicate whether a review protocol exists; state if and where it can be accessed (e.g., a Web address); and if available, provide registration information, including the registration number.	pg.15
Eligibility criteria	6	Specify characteristics of the sources of evidence used as eligibility criteria (e.g., years considered, language, and publication status), and provide a rationale.	pg.15-16 and S3
Information sources*	7	Describe all information sources in the search (e.g., databases with dates of coverage and contact with authors to identify additional sources), as well as the date the most recent search was executed.	pg.15
Search	8	Present the full electronic search strategy for at least 1 database, including any limits used, such that it could be repeated.	pg.15 and S2
Selection of sources of evidence†	9	State the process for selecting sources of evidence (i.e., screening and eligibility) included in the scoping review.	pg.15-16 and S3
Data charting process‡	10	Describe the methods of charting data from the included sources of evidence (e.g., calibrated forms or forms that have been tested by the team before their use, and whether data charting was done independently or in duplicate) and any processes for obtaining and confirming data from investigators.	pg.17-18
Data items	11	List and define all variables for which data were sought and any assumptions and simplifications made.	pg.17-18

SECTION	ITEM	PRISMA-ScR CHECKLIST ITEM	REPORTED ON PAGE #
Critical appraisal of individual sources of evidence§	12	If done, provide a rationale for conducting a critical appraisal of included sources of evidence; describe the methods used and how this information was used in any data synthesis (if appropriate).	N/A
Synthesis of results	13	Describe the methods of handling and summarizing the data that were charted.	pg.17-18
**RESULTS**			
Selection of sources of evidence	14	Give numbers of sources of evidence screened, assessed for eligibility, and included in the review, with reasons for exclusions at each stage, ideally using a flow diagram.	pg.4 and Figure 1
Characteristics of sources of evidence	15	For each source of evidence, present characteristics for which data were charted and provide the citations.	pg.4-6, Table 1 and Supplementary Data
Critical appraisal within sources of evidence	16	If done, present data on critical appraisal of included sources of evidence (see item 12).	N/A
Results of individual sources of evidence	17	For each included source of evidence, present the relevant data that were charted that relate to the review questions and objectives.	pg.4-6, Table 1 and Supplementary Materials
Synthesis of results	18	Summarize and/or present the charting results as they relate to the review questions and objectives.	pg.4-9
**DISCUSSION**			
Summary of evidence	19	Summarize the main results (including an overview of concepts, themes, and types of evidence available), link to the review questions and objectives, and consider the relevance to key groups.	pg.9-15
Limitations	20	Discuss the limitations of the scoping review process.	pg.13-14
Conclusions	21	Provide a general interpretation of the results with respect to the review questions and objectives, as well as potential implications and/or next steps.	pg.14-15
**FUNDING**			
Funding	22	Describe sources of funding for the included sources of evidence, as well as sources of funding for the scoping review. Describe the role of the funders of the scoping review.	pg.18

Table S2: Search strategy.

This table demonstrates the searches performed across the six databases and the search strategy for each.

**Database**	**Initial Search**		**Updated Search (from 2022 onwards)**	
	**Date of Search**	**Nº of Studies Identified**	**Date of Search**	**Nº of Studies Identified**
Ovid MEDLINE(R)	28.07.2022	1982	24.02.2025	630
CINAHL	02.08.2022	2482	24.02.2025	492
EBM REVIEWS	28.07.2022	286	24.02.2025	636
Embase Classic + Embase	28.07.2022	4662	24.02.2025	2371
Ovid Emcare	28.07.2022	1705	24.02.2025	1218
APA PsycInfo	28.07.2022	440	24.02.2025	126
**Total Studies (With Duplicates)**		11557		5473
**Total Studies (Without Duplicates)**		6953 (+1 expert identified)<br>• Duplicate records removed manually (n = 3352)<br>• Duplicate records removed by automation tools (n = 1252)		3287<br>• Duplicate records removed manually (n = 2039)<br>• Duplicate records removed by automation tools (n = 147)

Ovid MEDLINE (R)<br>**Host:** Ovid<br>**Date of Search:** Initial Search: 28.07.2022, Updated Search: 25.02.2025
**Search strategy**
1. Exp Pregnancy/
2. Exp Pregnancy Complications/
3. Exp Prenatal Care/
4. (antenatal or prenatal or pregnan*).ti,ab.
5. (childbear* adj2 wom*n).ti,ab.
6. Exp postpartum period/
7. Exp lactation/
8. (postpartum or postnatal or puerperium or lactating or "nursing women" or breastfeeding or "after birth" or "following pregnancy" or postpregnancy or "post pregnancy" or "following childbirth" or postpartal or "post-partal" or lactation or "breast-feeding" or "following birth" or "after delivery" or "post childbirth" or "post-partum" or "post-natal" or "post–pregnancy").ti,ab.
9. 1 OR 2 OR 3 OR 4 OR 5 OR 6 OR 7 OR 8
10. Exp interview/
11. Exp qualitative research/
12. ((semi-structured or semistructured or unstructured or informal or in-depth or indepth or face-to-face or structured or guide*) adj3 (interview* or discussion*)).ti,ab.
13. (“focus group*” or qualitative or “mixed method*” or “participant observ*” or “case stud*” or case-stud*).ti,ab.
14. 10 OR 11 OR 12 OR 13
15. Exp hypertension, pregnancy-induced/
16. ((Hypertensi* disorder*) adj2 pregnan*).ti,ab.

17. (preeclampsia or pre-eclampsia OR eclampsia or HDP or pregnancy-induced hypertension or pregnancy induced hypertension or gestational hypertension or high blood pressure in pregnancy).ti,ab.
18. 15 OR 16 OR 17
19. exp Fetal Growth Retardation/
20. Exp "Infant, Extremely Low Birth Weight"/
21. Exp "Infant, Very Low Birth Weight"/
22. Exp "Infant, Low Birth Weight"/
23. exp Infant, Small for Gestational Age/
24. (Growth restrict* adj2 (infant or f?et*)).ti,ab.
25. (Growth retard* adj2 (infant or f?et*)).ti,ab.
26. (IUGR or intrauterine growth restriction or low birth weight or SGA or small for gestational age or f?etal growth restriction or intrauterine growth retardation or low birth-weight infant* or very low birth weight infant*).ti,ab.
27. 19 OR 20 OR 21 OR 22 OR 23 OR 24 OR 25 OR 26
28. exp Infant, Premature/
29. exp Infant, Extremely Premature/
30. exp Labor Premature/
31. exp Obstetric Labor, Premature/
32. exp Premature Birth/
33. (pre?term adj2 (birth* or infant* or deliver* or rupture of membrane* or labo?r)).ti,ab.
34. (pre?mature adj2 (birth* or infant* or deliver* or rupture of membrane* or labo?r)).ti,ab.
35. (SPTB or PTB or neonatal prematur*or PROM or PPROM or pre?maturity).ti,ab.
36. 28 OR 29 OR 30 OR 31 OR 32 OR 33 OR 34 OR 35
37. exp Diabetes, Gestational/
38. ("gestational diabetes" or GDM or "pregnancy-induced diabetes" or "abnormal glucose tolerance").ti,ab.
39. 37 OR 38
40. 18 OR 27 OR 36 OR 39
41. 9 AND 14 AND 40
42. limit 40 to humans
CINAHL Plus<br>**Host:** EBSCO<br>**Date of Search:** Initial Search: 02.08.2022, Updated Search: 25.02.2025
**Search strategy**
S1. MH "Pregnancy+"
S2. TI (antenatal or prenatal or pregnan* or (childbear* N2 wom#n)) OR AB (antenatal or prenatal or pregnan* or (childbear* N2 wom#n))
S3. (MH "Prenatal Care+")
S4. (MH "Postnatal Period+")
S5. TI (postpartum or postnatal or puerperium or lactating or "nursing women" or breastfeeding or "after birth" or "following pregnancy" or postpregnancy or "post pregnancy" or "following childbirth" or postpartal or "post-partal" or lactation or "breast-feeding" or "following birth" or "after delivery" or "post childbirth" or "post-partum" or "post-natal" or "post–pregnancy") or AB (postpartum or postnatal or puerperium or lactating or "nursing women" or breastfeeding or "after birth" or "following pregnancy" or postpregnancy or "post pregnancy" or "following childbirth" or postpartal or "post-partal" or lactation or "breast-feeding" or "following birth" or "after delivery" or "post childbirth" or "post-partum" or "post-natal" or "post–pregnancy")
S6. S1 OR S2 OR S3 OR S4 OR S5
S7. (MH "Exit Interviews") OR (MH "Interviews+") OR (MH "Focus Groups")

S8. (MH "Qualitative Studies+")
S9. TI ((semi-structured or semistructured or unstructured or informal or in-depth or indepth or face-to-face or structured or guide) N3 (interview* or discussion*)) or AB ((semi-structured or semistructured or unstructured or informal or in-depth or indepth or face-to-face or structured or guide*) N3 (interview* or discussion*))
S10. TI (“focus group*” or qualitative or “mixed method*” or “participant observ*” or “case stud*” or case-stud*) or AB (“focus group*” or qualitative or “mixed method*” or “participant observ*” or “case stud*” or “case-stud*)
S11. S7 OR S8 OR S9 OR S10
S12. (MH "Pregnancy-Induced Hypertension+")
S13. TI ("hypertensive disorder*" of pregnancy or "hypertension in pregnancy" or preeclampsia or pre-eclampsia or eclampsia or HDP or maternal hypertension or "pregnancy-induced hypertension" or "pregnancy induced hypertension" or "gestational hypertension" or "high blood pressure in pregnancy") or AB ("hypertensive disorder*" of pregnancy or "hypertension in pregnancy" or preeclampsia or pre-eclampsia or eclampsia or HDP or maternal hypertension or "pregnancy-induced hypertension" or "pregnancy induced hypertension" or "gestational hypertension" or "high blood pressure in pregnancy")
S14. S12 OR S13
S15. (MH "Fetal Growth Retardation")
S16. (MH "Infant, Low Birth Weight+")
S17. TI (IUGR or "intrauterine growth restriction" or "low birth weight" or SGA or "small for gestational age" or "f?etal growth restriction" or "intrauterine growth retardation”) or AB (IUGR or "intrauterine growth restriction" or "low birth weight" or SGA or "small for gestational age" or "f?etal growth restriction" or "intrauterine growth retardation”)
S18. S15 OR S16 OR S17
S19. (MH "Infant, Premature")
S20. (MH "Childbirth, Premature")
S21. (MH "Labor, Premature")
S22. TI (pre#term N2 (birth* or infant* or deliver* or rupture of membrane* or labo#r or "Obstetric Labour")) or AB (pre#term N2 (birth* or infant* or deliver* or rupture of membrane* or labo#r or "Obstetric Labour"))
S23. TI (pre#mature N2 (birth* or infant* or deliver* or rupture of membrane* or labo#r or "Obstetric Labour")) or AB (pre#mature N2 (birth* or infant* or deliver* or rupture of membrane* or labo#r or "Obstetric Labour"))
S24. TI (SPTB or PTB or neonatal prematur*or PROM or PPROM or pre?maturity) or AB (SPTB or PTB or neonatal prematur*or PROM or PPROM or pre?maturity)
S25. S19 OR S20 OR S21 OR S22 OR S23 OR S24
S26. (MH "Diabetes Mellitus, Gestational+")
S27. TI ("gestational diabetes" or GDM or "pregnancy-induced diabetes" or "abnormal glucose tolerance") or AB ("gestational diabetes" or GDM or "pregnancy-induced diabetes" or "abnormal glucose tolerance")
S28. S26 or S27
S29. S14 OR S18 OR S25 OR S28
S30. S6 AND S11 AND S29
EMBASE Classic + EMBASE<br>**Host:** Ovid<br>**Date of Search:** Initial Search: 28.07.2022, Updated Search: 25.02.2025
**Search strategy**
1. Exp Pregnancy/
2. Exp High Risk Pregnancy/

3. Exp Pregnancy Complication/
4. Exp Prenatal Care/
5. (antenatal or prenatal or pregnan*).ti,ab.
6. (childbear* adj2 wom*n).ti,ab.
7. Exp Puerperium/
8. Exp Lactation/
9. (postpartum or postnatal or puerperium or lactating or "nursing women" or breastfeeding or "after birth" or "following pregnancy" or postpregnancy or "post pregnancy" or "following childbirth" or postpartal or "post-partal" or lactation or "breast-feeding" or "following birth" or "after delivery" or "post childbirth" or "post-partum" or "post-natal" or "post–pregnancy").ti,ab.
10. 1 OR 2 OR 3 OR 4 OR 5 OR 6 OR 7 OR 8 OR 9
11. Exp Interview/
12. Exp Qualitative Research/
13. ((semi-structured or semistructured or unstructured or informal or in-depth or indepth or face-to-face or structured or guide*) adj3 (interview* or discussion*)).ti,ab.
14. ("focus group*" or qualitative or "mixed method*" or "participant observ*" or "case stud*" or case-stud*).ti,ab.
15. 11 OR 12 OR 13 OR 14
16. Exp Maternal Hypertension/
17. ((Hypertensi* disorder*) adj2 pregnan*).ti,ab.
18. (preeclampsia or pre-eclampsia OR eclampsia or HDP or pregnancy-induced hypertension or pregnancy induced hypertension or gestational hypertension or high blood pressure in pregnancy).ti,ab.
19. 16 OR 17 OR 18
20. Exp Intrauterine Growth Retardation/
21. Exp Low Birth Weight/
22. Exp Small for Date Infant/
23. (Growth restrict* adj2 (infant or f?et*)).ti,ab.
24. (Growth retard* adj2 (infant or f?et*)).ti,ab.
25. (IUGR or intrauterine growth restriction or low birth weight or SGA or small for gestational age or f?etal growth restriction or intrauterine growth retardation or low birth-weight infant* or very low birth weight infant*).ti,ab.
26. 20 OR 21 OR 22 OR 23 OR 24 OR 25
27. Exp Prematurity/
28. Exp "Immature and Premature Labor"/
29. Exp Premature Labor/
30. (pre?term adj2 (birth* or infant* or deliver* or rupture of membrane* or labo?r)).ti,ab.
31. (pre?mature adj2 (birth* or infant* or deliver* or rupture of membrane* or labo?r)).ti,ab.
32. (SPTB or PTB or neonatal prematur*or PROM or PPROM or pre?maturity).ti,ab.
33. 27 OR 28 OR 29 OR 30 OR 30 OR 31 OR 32
34. Exp Pregnancy Diabetes Mellitus/
35. ("gestational diabetes" or GDM or "pregnancy-induced diabetes" or "abnormal glucose tolerance").ti,ab.
36. 34 OR 35
37. 19 OR 26 OR 33 OR 36
38. 10 AND 15 AND 37
39. limit 38 to humans

APA PsycInfo<br>**Host:** Ovid<br>**Date of Search:** Initial Search: 28.07.2022, Updated Search: 25.02.2025
**Search strategy**
1. Exp Pregnancy/
2. Exp Prenatal Care/
3. (antenatal or prenatal or pregnan*).ti,ab.
4. (childbear* adj2 wom*n).ti,ab.
5. Exp lactation/
6. (postpartum or postnatal or puerperium or lactating or "nursing women" or breastfeeding or "after birth" or "following pregnancy" or postpregnancy or "post pregnancy" or "following childbirth" or postpartal or "post-partal" or lactation or "breast-feeding" or "following birth" or "after delivery" or "post childbirth" or "post-partum" or "post-natal" or "post–pregnancy").ti,ab.
7. 1 OR 2 OR 3 OR 4 OR 5 OR 6
8. Exp interviews/
9. Exp qualitative methods/
10. ((semi-structured or semistructured or unstructured or informal or in-depth or indepth or face-to-face or structured or guide*) adj3 (interview* or discussion*)).ti,ab.
11. (“focus group*” or qualitative or “mixed method*” or “participant observ*” or “case stud*” or case-stud*).ti,ab.
12. 8 OR 9 OR 10 OR 11
13. Exp preeclampsia/
14. ((Hypertensi* disorder*) adj2 pregnan*).ti,ab.
15. (preeclampsia or pre-eclampsia OR eclampsia or HDP or pregnancy-induced hypertension or pregnancy induced hypertension or gestational hypertension or high blood pressure in pregnancy).ti,ab.
16. 13 OR 14 OR 15
17. (IUGR or intrauterine growth restriction or low birth weight or SGA or small for gestational age or f?etal growth restriction or intrauterine growth retardation or low birth-weight infant* or very low birth weight infant*).ti,ab.
18. exp birth weight/
19. exp Premature Birth/
20. (pre?term adj2 (birth* or infant* or deliver* or rupture of membrane* or labo?r)).ti,ab.
21. (pre?mature adj2 (birth* or infant* or deliver* or rupture of membrane* or labo?r)).ti,ab.
22. (SPTB or PTB or neonatal prematur*or PROM or PPROM or pre?maturity).ti,ab.
23. 18 or 19 or 20 or 21 or 22
24. exp Gestational Diabetes/
25. ("gestational diabetes" or GDM or "pregnancy-induced diabetes" or "abnormal glucose tolerance").ti,ab.
26. 24 OR 25
27. 16 OR 17 OR 23 OR 26
28. 7 AND 12 AND 27
29. limit 28 to humans
**Ovid Emcare**<br>**Host:** Ovid<br>**Date of Search:** Initial Search: 28.07.2022, Updated Search: 25.02.2025
**Search strategy**
1. Exp Pregnancy/

2. Exp high risk pregnancy/
3. Exp pregnancy complication/
4. Exp Prenatal Care/
5. (antenatal or prenatal or pregnan*).ti,ab.
6. (childbear* adj2 wom*n).ti,ab.
7. Exp puerperium/
8. Exp lactation/
9. (postpartum or postnatal or puerperium or lactating or "nursing women" or breastfeeding or "after birth" or "following pregnancy" or postpregnancy or "post pregnancy" or "following childbirth" or postpartal or "post-partal" or lactation or "breast-feeding" or "following birth" or "after delivery" or "post childbirth" or "post-partum" or "post-natal" or "post–pregnancy").ti,ab.
10. 1 OR 2 OR 3 OR 4 OR 5 OR 6 OR 7 OR 8 OR 9
11. Exp interview/
12. Exp qualitative research/
13. ((semi-structured or semistructured or unstructured or informal or in-depth or indepth or face-to-face or structured or guide*) adj3 (interview* or discussion*)).ti,ab.
14. ("focus group*" or qualitative or "mixed method*" or "participant observ*" or "case stud*" or "case-stud*").ti,ab.
15. 11 OR 12 OR 13 OR 14
16. Exp hypertension, pregnancy-induced/
17. ((Hypertensi* disorder*) adj2 pregnan*).ti,ab.
18. (preeclampsia or pre-eclampsia OR eclampsia or HDP or pregnancy-induced hypertension or pregnancy induced hypertension or gestational hypertension or high blood pressure in pregnancy).ti,ab.
19. 16 OR 17 OR 18
20. Exp Fetal Growth Retardation/
21. Exp "Infant, Extremely Low Birth Weight"/
22. Exp "Infant, Very Low Birth Weight"/
23. Exp "Infant, Low Birth Weight"/
24. Exp Infant, Small for Gestational Age/
25. (Growth restrict* adj2 (infant or f?et*)).ti,ab.
26. (Growth retard* adj2 (infant or f?et*)).ti,ab.
27. (IUGR or intrauterine growth restriction or low birth weight or SGA or small for gestational age or f?etal growth restriction or intrauterine growth retardation or low birth-weight infant* or very low birth weight infant*).ti,ab.
28. 20 OR 21 OR 22 OR 23 OR 24 OR 25 OR 26 OR 27
29. exp Infant, Premature/
30. exp Infant, Extremely Premature/
31. exp Labor Premature/
32. exp Obstetric Labor, Premature/
33. exp Premature Birth/
34. (pre?term adj2 (birth* or infant* or deliver* or rupture of membrane* or labo?r)).ti,ab.
35. (pre?mature adj2 (birth* or infant* or deliver* or rupture of membrane* or labo?r)).ti,ab.
36. (SPTB or PTB or neonatal prematur*or PROM or PPROM or pre?maturity).ti,ab.
37. 29 OR 30 OR 31 OR 32 OR 33 OR 34 OR 35 OR 36
38. Exp pregnancy diabetes mellitus/
39. ("gestational diabetes" or GDM or "pregnancy-induced diabetes" or "abnormal glucose tolerance").ti,ab.
40. 38 or 39
41. 19 OR 28 OR 37 OR 40

42. 10 AND 15 AND 41
43. limit 42 to humans
All EBM Reviews – Cochrane DSR, ACP Journal Club, DARE, CCA, CCTR, CMR, HTA, and NHSEED<br>**Host:** Ovid<br>**Date of Search:** Initial Search: 28.07.2022, Updated Search: 25.02.2025<br>**Databases:**<br>• EBM Reviews - Cochrane Database of Systematic Reviews 2005 to July 27, 2022<br>• EBM Reviews - ACP Journal Club 1991 to July 2022<br>• EBM Reviews - Database of Abstracts of Reviews of Effects 1st Quarter 2016<br>• EBM Reviews - Cochrane Clinical Answers July 2022<br>• EBM Reviews - Cochrane Central Register of Controlled Trials June 2022<br>• EBM Reviews - Cochrane Methodology Register 3rd Quarter 2012<br>• EBM Reviews - Health Technology Assessment 4th Quarter 2016<br>• EBM Reviews - NHS Economic Evaluation Database 1st Quarter 2016
**Search strategy**
1. Exp Pregnancy/
2. Exp High Risk Pregnancy/
3. Exp Pregnancy Complication/
4. Exp Prenatal Care/
5. (antenatal or prenatal or pregnan*).ti,ab.
6. (childbear* adj2 wom*n).ti,ab.
7. Exp postpartum period/
8. Exp lactation/
9. (postpartum or postnatal or puerperium or lactating or "nursing women" or breastfeeding or "after birth" or "following pregnancy" or postpregnancy or "post pregnancy" or "following childbirth" or postpartal or "post-partal" or lactation or "breast-feeding" or "following birth" or "after delivery" or "post childbirth" or "post-partum" or "post-natal" or "post–pregnancy").ti,ab.
10. 1 OR 2 OR 3 OR 4 OR 5 OR 6 OR 7 OR 8 OR 9
11. Exp interview/
12. Exp qualitative research/
13. ((semi-structured or semistructured or unstructured or informal or in-depth or indepth or face-to-face or structured or guide*) adj3 (interview* or discussion*)).ti,ab.
14. (“focus group*” or qualitative or “mixed method*” or observ* or “case stud*” or case-stud*).ti,ab.
15. 11 OR 12 OR 13 OR 14
16. Exp hypertension, pregnancy-induced/
17. ((Hypertensi* disorder*) adj2 pregnan*).ti,ab.
18. (preeclampsia or pre-eclampsia OR eclampsia or HDP or pregnancy-induced hypertension or pregnancy induced hypertension or gestational hypertension or high blood pressure in pregnancy).ti,ab.
19. 16 OR 17 OR 18
20. exp Fetal Growth Retardation/
21. Exp "Infant, Extremely Low Birth Weight"/
22. Exp "Infant, Very Low Birth Weight"/
23. Exp "Infant, Low Birth Weight"/
24. exp Infant, Small for Gestational Age/
25. (Growth restrict* adj2 (infant or f?et*)).ti,ab.

26.	(Growth retard* adj2 (infant or f?et*)).ti,ab.
27.	(IUGR or intrauterine growth restriction or low birth weight or SGA or small for gestational age or f?etal growth restriction or intrauterine growth retardation or low birth-weight infant* or very low birth weight infant*).ti,ab.
28.	20 OR 21 OR 22 OR 23 OR 24 OR 25 OR 26 OR 27
29.	exp Infant, Premature/
30.	exp Infant, Extremely Premature/
31.	exp Labor Premature/
32.	exp Obstetric Labor, Premature/
33.	exp Premature Birth/
34.	(pre?term adj2 (birth* or infant* or deliver* or rupture of membrane* or labo?r)).ti,ab.
35.	(pre?mature adj2 (birth* or infant* or deliver* or rupture of membrane* or labo?r)).ti,ab.
36.	(SPTB or PTB or neonatal prematur*or PROM or PPROM or pre?maturity).ti,ab.
37.	29 OR 30 OR 31 OR 32 OR 33 OR 34 OR 35 OR 36
38.	exp Diabetes, Gestational/
39.	("gestational diabetes" or GDM or "pregnancy-induced diabetes" or "abnormal glucose tolerance").ti,ab.
40.	38 or 39
41.	19 OR 28 OR 37 OR 40
42.	10 AND 15 AND 41
43.	limit 42 to humans

Table S3: Inclusion and exclusion criteria.

This table demonstrates the inclusion and exclusion criteria used for the scoping review.

**SPIDER criteria**	**Inclusion criteria**	**Exclusion criteria**
Sample:	**Women:** Women at risk of GDM, HDP, FGR or PTB on an individual level based on any defined criteria or diagnosed during pregnancy or in the postpartum using any predetermined diagnostic criteria.<br>**Stakeholders:** Key stakeholders who describe women's experiences and engagement with care. Stakeholders include partners, healthcare professionals, other community stakeholders and other professional stakeholders.	**Women**: Women not at increased risk of or diagnosed with GDM, HDP, FGR or PTB. This includes women undergoing universal or routine screening.<br>**Stakeholders:** Stakeholder perspectives of universal screening, healthcare treatments or interventions without discussing women's engagement or views of this treatment.
Phenomenon of Interest:	**Phenomenon**: Women's experiences, viewpoints or attitudes towards their health/wellbeing and healthcare during pregnancy or postpartum, including breastfeeding/expressing breast milk outside of an acute care setting.<br>**Setting:** Perspectives of maternal health/wellbeing or healthcare within a hospital, acute care, outpatient, community, or home setting.	**Phenomenon**: Perspectives of the health of an infant or child, including counselling on the viability, potential health outcomes of a preterm infant and kangaroo care. Perspectives on policy development or implementation.<br>**Setting**: Perspectives of child or infant health or breastfeeding/expressing breast milk within an acute paediatric setting e.g., neonatal intensive care unit (NICU).
Design:	Focus group interviews, semi-structured interviews, in-depth questionnaires including open-ended qualitative questions, qualitative case studies, field observations/researcher notes and/or social media content analyses.	Quantitative research studies.
Evaluation:	N/A	N/A
Research type:	Qualitative data obtained from peer reviewed qualitative research studies, mixed method studies or review articles.	Grey literature and qualitative data published in books, theses, film/broadcast, opinion articles, narrative review articles, pre-prints, conference abstracts, abstracts with no full study available and protocol papers. Studies not accessible in the English language.

Abbreviations: FGR; fetal growth restriction, GDM; gestational diabetes mellitus, HDP; hypertensive disorders of pregnancy, NICU; neonatal intensive care unit, PTB; preterm birth; SPIDER; sample, phenomenon of interest, design, evaluation, and research type.

Table S4: Rationale for categorisation of studies.

This table demonstrates the rationale used for the categorisation of studies based on primary aim.

Category	***Primary Area of Investigation (Definition)***	***Rationale for creation of the category***
Prevention (Pregnancy)		
Prevention	Articles primarily explore women's experiences of medical, lifestyle	This category was developed to identify studies relating to *Prevention, Selective* and *Prevention,*

	(diet/activity) and/or pharmacotherapy interventions or behaviours to prevent the onset of cardiometabolic conditions during pregnancy.	*Indicative* as per the IOM Continuum of Care model.[1]
Treatment (Pregnancy)		
Screening and Diagnosis	Articles primarily explore women's experiences of screening or diagnosis during pregnancy. This includes women's understanding and perceived risk of the cardiometabolic pregnancy complication.	This category was developed to identify studies relating to *Treatment, Case identification* as per the IOM Continuum of Care model.[1]
Treatment and Management	Articles primarily explore women's experiences of medical, lifestyle (diet/activity) and/or pharmacotherapy interventions or behaviours during pregnancy. This includes medical consults, such as antenatal consults for preterm labour.	This category was developed to identify studies relating to *Treatment, Standard treatment for known disorders* as per the IOM Continuum of Care model.[1]
Psychosocial Health	Articles primarily explore psychological, emotional and/or social aspects of women's health during pregnancy.	This category was *iteratively developed* in response to the primary aims of eligible studies.
Healthcare Systems and Delivery	Articles primarily relate to functioning of the healthcare system and explore women's experiences of quality, distribution, or access to resources during pregnancy.	This category was developed in response to the implications/ recommendations outlined in Parikh et al.[2] American Heart Association Scientific Statement on Adverse Pregnancy Outcomes and Cardiovascular Disease Risk. This category seeks to identify studies that relate to the recommendation, "*Health care systems need to improve transitions of care for women with APOs and implement targeted healthcare strategies to reduce long-term CVD risk.*[2(pg.e911)]"
Maintenance (Postpartum)		
Screening for Cardiometabolic Conditions	Articles primarily explore women's experiences of screening for a chronic disease after pregnancy. This includes women's understanding and perceived risks of the condition.	This category was developed to identify studies relating to *Maintenance, Compliance with long-term treatment and reduction and risk reduction* as per the IOM Continuum of Care model.[1]<br><br>This category was developed in response to the implications/ recommendations outlined in Parikh et al.[2] American Heart Association Scientific Statement on Adverse Pregnancy Outcomes and Cardiovascular Disease Risk. This category seeks to identify studies that relate to the recommendation, *"Consideration of APOs is essential when evaluating CVD risk in women.*[2(e910)]*"*
Optimizing Maternal Health	Articles primarily explore women's experiences of medical, healthy lifestyles (diet/activity) and/or pharmacotherapy interventions or behaviours to optimize general or cardiometabolic health, including in relation to cardiometabolic	This category was developed to identify studies relating to *Maintenance, Compliance with long-term treatment and reduction and risk reduction* as per the IOM Continuum of Care model.[1]<br><br>This category was developed in response to the implications/ recommendations outlined in

	complications in the postpartum period.	Parikh et al.[2] American Heart Association Scientific Statement on Adverse Pregnancy Outcomes and Cardiovascular Disease Risk. This category seeks to identify studies that relate to the recommendation, *"Adopting a heart-healthy diet and increasing physical activity among women with APOs, starting in the postpartum setting and continuing across the life span, are important lifestyle interventions to decrease CVD risk,*[2(e910)]*"* and *"Studies of aspirin, statins, and metformin may better inform our recommendations for pharmacotherapy in primary CVD prevention among women who have had an APO.*[2(e911)]*"*
Breastfeeding	Articles primarily explore women's experiences of breastfeeding.	This category was developed to identify studies relating to *Maintenance, Compliance with long-term treatment and reduction and risk reduction* as per the IOM Continuum of Care model.[1]<br><br>This category was developed in response to the implications/ recommendations outlined in Parikh et al.[2] American Heart Association Scientific Statement on Adverse Pregnancy Outcomes and Cardiovascular Disease Risk. This category seeks to identify studies that relate to the recommendation, "*Lactation and breastfeeding may lower a woman's later cardiometabolic risk.*[2(e911)]*"*
Healthcare Systems and Delivery	Articles primarily relate to the functioning of the healthcare system and explore women's experiences of quality, distribution, or access to resources in the postpartum.	This category was developed in response to the implications/ recommendations outlined in Parikh et al.[2] American Heart Association Scientific Statement on Adverse Pregnancy Outcomes and Cardiovascular Disease Risk. This category seeks to identify studies that relate to the recommendation, "*Health care systems need to improve transitions of care for women with APOs and implement targeted healthcare strategies to reduce long-term CVD risk.*[2(e911)]*"*
Psychosocial Health	Articles primarily explore psychological, emotional and/or social aspects of women's health in the postpartum, including women's experiences of parenting after a cardiometabolic complication of pregnancy.	This category was *iteratively developed* in response to the primary aims of eligible studies.

Abbreviations: APO; adverse pregnancy outcome, CVD; cardiovascular disease, IOM; Institute of Medicine.

Table S5: Summary of Leximancer analysis settings (variations to default settings) used to generate concept maps.

This table shows a summary of the Leximancer analysis settings (variations to default settings) used to generate concept maps.

	Gestational Diabetes Mellitus	Hypertensive Disorders of Pregnancy	Preterm Birth	Fetal growth restriction
**Network type**	Social network (Gaussian)	Social network (Gaussian)	Social network (Gaussian)	Social network (Gaussian)
**Text processing**	1 sentence per block	1 sentence per block	1 sentence per block	1 sentence per block
**Concept Seed – Merged**	Diet / dietary<br>Health / healthy<br>Intervention / interventions<br>Need / needs<br>Professionals / providers<br>Women / participants	Experience / experiences<br>Need / needs<br>Participants / patients / women<br>Pre-eclampsia / preeclampsia<br>Pregnancy / pregnant<br>Professionals / providers	Baby / babies / child / infant<br>Experience / experiences<br>Mother / mothers/ maternal / participant / women / women's<br>Need / needs<br>Premature / preterm / PTB<br>Professionals / providers	Hospital / hospitalization<br>Child / infant / infants<br>Mothers / participants / woman /women<br>Participating / participation<br>Pregnancy / pregnant
**Concept Seeds – Deleted**	During<br>Emerged<br>Factors<br>Findings<br>Identified<br>Included<br>Including<br>Related<br>Reported<br>Study<br>Themes<br>Use	Analysis<br>During<br>Factors<br>Groups<br>Identified<br>Included<br>Including<br>Making<br>Regarding<br>Reported<br>Study<br>Themes<br>Use<br>Used	Analysis<br>Associated<br>Described<br>During<br>Emerged<br>Findings<br>Identified<br>Included<br>Main<br>Regarding<br>Reported<br>Revealed<br>Study<br>Theme<br>Themes	Analysis<br>Described<br>Experienced<br>Identified<br>Participating<br>Participation<br>Provided<br>Reported<br>Research<br>Sources<br>Study<br>Theme<br>Themes
**Theme Size**	60%	60%	60%	60%
**Visible Concepts**	100%	100%	100%	100%

References

1. Institute of Medicine Committee on Prevention of Mental Disorders. *Reducing Risks for Mental Disorders: Frontiers for Preventive Intervention Research.* National Academies Press; 1994.
2. Parikh NI, Gonzalez JM, Anderson CAM, et al. Adverse Pregnancy Outcomes and Cardiovascular Disease Risk: Unique Opportunities for Cardiovascular Disease Prevention in Women: A Scientific Statement From the American Heart Association. *Circulation.* 2021;143(18):e902-e916.
